# A Comparative Study of Classroom and Online Distance Modes of Official Vocational Education and Training

**DOI:** 10.1371/journal.pone.0096052

**Published:** 2014-05-01

**Authors:** Miguel Vicente López Soblechero, Cristina González Gaya, Juan José Hernández Ramírez

**Affiliations:** 1 Escuela Técnica Superior de Ingenieros Industriales, Universidad Nacional de Educación a Distancia (UNED), Madrid, Spain; 2 IES Alonso de Avellaneda, Consejería de Educación de la Comunidad de Madrid, Alcalá de Henares, Madrid, Spain; University of Westminster, United Kingdom

## Abstract

The study discussed in this paper had two principal objectives. The first was to evaluate the distance model of official vocational education and training offered by means of a virtual learning platform. The second was to establish that both on-site classroom and online distance modes of vocational education and training can be seen as complementary in terms of responding to the majority of modern educational needs. We performed a comparative study using data and results gathered over the course of eleven academic years for 1,133 of our students enrolled in an official vocational education and training program, leading to the awarding of a certificate as an Administrative Management Expert. The classes were offered by the Alfonso de Avellaneda Vocational Education and Training School, located in the city of Alcalá de Henares near Madrid, Spain. We offered classes both in traditional classroom mode and through online distance learning. This paper begins with a descriptive analysis of the variables we studied; inferential statistical techniques are subsequently applied in order to study the relationships that help form the basis for the conclusions reached. This study’s results provide evidence that a broad offering of vocational education and training opportunities will facilitate access to such learning for students who require it, regardless of their age, employment status, or personal circumstances, with the online distance mode playing a fundamental role while also yielding results equivalent to those observed for classroom instruction.

## Introduction

In Spain’s current economic situation, where the unemployment rate for workers with relatively low qualifications has reached 41.2%, and where 47% of the working-age population has a lower secondary education level or less, it is clear that the youngest job seekers as well as unemployed workers with insufficient qualifications must receive further education in order to succeed in the workplace. Vocational education and training opportunities have therefore emerged as a key factor in terms of the country’s economic recovery [1]–[2].

Various reports produced by the European Centre for the Development of Vocational Training (known as Cedefop from its acronym in French) indicate that the employment offered throughout the European Union between now and 2020 will change in terms of the specializations demanded. The number of low-skill jobs will also decline because of higher levels of specialization in industry, technological advances, and increasing automation of production processes, among other factors [3]–[4]. Changes in the production model, high rates of unemployment, and the fact that many Spanish workers have only low levels of formal education are some of the factors that have played an influential role in the greatly increased demand for vocational education and training seen in Spain in recent years. To the extent that Spain’s economic crisis situation continues to improve, the need for workers qualified in the new specializations the job market will be demanding means that online distance vocational education will play a fundamental role, both because of the flexibility it offers as well as its ability to reach the largest number of students at lower costs compared to on-site classroom teaching.

The educational level of a country’s population not only conditions its possibilities for development but influences other factors as well. For example, previous studies have found relationships between a population’s educational level and certain types of health problems [5]–[9]. Education has become fundamental in multiple aspects of our society and it is no longer thought of as a temporary stage ending in the granting of a degree, but instead as a life-long, ongoing process. There are also a diversity of activities supported by new technologies that can help delay the onset of certain types of problems associated with age in retired workers, thereby improving their quality of life [10]–[12]. Education will continue to be a part of all our lives in one way or another and at least a portion of it will be provided via an online virtual platform.

Various published studies have discussed the benefits associated with the use of virtual learning platforms [13]–[15]. For example, Heaperman and Sudweeks [16] believe that using a virtual platform in any distance learning system has become indispensible. Virtual learning platforms for education have already changed the manner in which teaching is offered and learning takes place. Heaton-Shrestha et al. [17] have emphasized the impact of an online e-learning platform on teaching practices. Queiroz and Mustaro [18] and Awouters and Jans [19] have conducted studies on the new skills that teachers must acquire in order to provide added value through the use of online platforms. It is clear that teachers and professors must receive the appropriate types of training in order to ensure that the teaching and learning processes can take place in the most effective manner possible when supported by a virtual learning environment. Santos-Rego et al. [20] have also emphasized the need for teaching professionals to be sufficiently prepared in order to take full advantage of CIT-based approaches (Communications and Information Technology).

Students on the other hand, especially the youngest ones, are already accustomed to using computers, mobile phones, and tablets to search for all types of information, for purposes of entertainment, communication, discussions in online forums, etc. This puts them at an advantage in terms of learning how to use a virtual classroom system almost immediately. However, their extensive use of high-tech devices in their free time does not always translate into improved academic results [21].

Regardless of the type of platform, tools, or resources employed, the pedagogical aspect will always remain fundamental, as emphasized by Garcia-Aretio [22]. It is important for instructors to actively encourage students to participate in the activities and forums designed to promote interaction, in order to prevent a loss of motivation and interest. Wessa et al. [23] have also found that students with the highest participation levels, who respond to the most messages, increase their probability of obtaining the best results.

Bri et al. [24] have suggested that experts in teaching methodologies should design the contents of an online learning environment by taking into account aspects such as the quality and quantity of information incorporated, the level of interactivity, and the platform’s appropriateness for the needs and capabilities of the students. An online platform should also be user-friendly and accessible, so that any instructor can create and add content to it without the need to become a computer expert. As recommended by Villalustre and Del Moral [25], it is also important to take student learning styles and student preferences into account when creating content and developing activities. Versatility is the aspect of a virtual learning platform that allows easy adaptation of the contents to the specific characteristics of the subject being taught and to the group of students who will be using it. Ríos-Guardiola [26] has analyzed the advantages and limitations of some of the most commonly used tools offering virtual learning environments. Scientific and technological advances represent a challenge for scientists as well as professors, who must stay up-to-date in their respective specializations. New information and materials are constantly being published online and made available on the web, as discussed by Kerfeld and Gross [27]. The flexibility of a virtual learning environment allows its contents to be expanded and updated in a simple and rapid manner.

Among the virtual platforms available on a worldwide basis, Moodle is the most widely distributed freeware virtual learning platform, while Blackboard is one of the most commonly used packages requiring software to be purchased. Although there are some existing publications on the results of comparative studies focused on application of various online teaching platforms applied in the university setting, equivalent studies focused on vocational education and training have not yet begun to appear. Bremer and Bryant [28] have presented a comparison of the two leading platforms (Moodle and Blackboard) from the point of view of students, teachers, and platform administrators. In addition, aspects such as functionality, user preferences, interface characteristics, and efficacy have been comparatively analyzed by Machado and Tao [29], who concluded that the Moodle platform was more efficient and effective than Blackboard. Other studies have also pointed out that many institutions have been switching from platforms where software must be purchased to freeware platforms, with most of these changes being made to Moodle [30].

In the case being discussed here, Moodle was the platform selected, since in addition to any advantages it may confer, it is the one the Community of Madrid Educational Council provides to schools and learning centers under its supervision. All online teaching content developed by the Council itself also currently uses Moodle. Other vocational education and training centers have gradually but unevenly been adopting this tool, mainly as a means of support for classroom teaching. At the time when the Alonso de Avellaneda Vocational Education and Training School began using Moodle for its distance learning Administrative Management program, we were the first school in the Community of Madrid to employ this tool to present an entirely distance-mode vocational education program and for this reason, our center now is most experienced in the area. During the 2000–2001 academic year, the program offered morning-early afternoon and late afternoon-evening classes with a different group of students enrolled in each of these. Later, during the 2002–2003 year, a second morning-early afternoon group was added. The distance-mode teaching program was first implemented during the 2004–2005 academic year.

For the 2011–2012 academic year, a new updated curriculum was implemented for the vocational program in all modalities. The contents and structure of assignments were modified along with the number of teaching hours.

To carry out the objectives developed for this study, data has been analyzed for students enrolled in both the classroom and online distance modality, throughout the eleven academic years from 2000–2011, at the Alonso de Avellaneda Vocational Education and Training School in the city of Alcalá de Henares near Madrid. The conclusions drawn based upon this data confirms that importance and validity of the vocational education and training system under study.

## Materials and Methods

During the eleven academic years represented by the period studied, 1,133 students enrolled in the Administrative Management vocational program during the morning-early afternoon or late afternoon-evening classes or through online distance learning program. The results obtained by all of these students in the eight subjects incorporated into the vocational program were studied along with the students’ ages and gender. A series of surveys was also given to expand the data sets as well as to evaluate the online learning platform. Using this data, information related to the following variables was compiled for each student:

Year: indicates the year in which the student completed the training program.
Subject: eight variables recorded, one for each subject.
Classes: classes attended by the student (morning-early afternoon, lateafternoon-evening, or distance).
Gender: male or female.
Age.
Group.

Another set of variables was defined based on the above data sets in order to expand the scope of the study:

Average grade received.
Number of subjects passed.
Number of subjects failed.
Number of subjects in which the student formally withdrew from the subject before exam time.
Number of subjects where the student did not attend the exams.
Advancement: indicates whether or not the student advanced to the next level.
Withdrawal: indicates whether the student withdrew from the academic program.

The dataset is attached as a supporting information file (Table S1).

Quantitative results were obtained for each of the variables: mean, median, mode, standard deviation, variance, skewness, kurtosis, range, minimum, maximum, and percentiles. The frequency distributions were also analyzed. Prior to selecting the statistical tests to be employed for a detailed comparative analysis, the following issues were taken into consideration. First, the data obtained for each of the student groups were independent, since the membership of each of the class groups (morning-early afternoon, late afternoon-evening, or distance) was different. In terms of the academic years, these were also independent samples since each year was independent from the next.

The normality study for the variables was conducted using the following set of contrasting hypotheses, based upon the significance level obtained by the Kolmogorov-Smirnov test:

Null hypothesis (H0): the distribution of data for a variable follows a normal distribution
Alternative hypothesis (H1): the distribution of data for a variable *does not* follow a normal distribution

The significance level (*p*) selected was 0.05. Therefore, if the significance test obtained *p*<0.05 (5%), the null hypothesis was rejected (H0) and it was concluded that the variable did not follow a normal distribution. On the other hand, for significance levels where *p*>0.05, the H0 was accepted and it was concluded that the variable’s data followed a normal distribution.

The results of this normality testing for the variables showed that only the average grade received variable for students in the late afternoon-evening class showed a normal distribution. The box plots also show that, for most of the variables, there is an abundance of anomalous observations. This means that the conditions necessary for application of Student’s t-test or ANOVA were not present, and we were therefore required to use a non-parametric test. The non-parametric tests employed in order to study possible differences among the groups were the Mann-Whitney test and the Kruskall-Wallis test. The first was used when the groups were studied two at a time and the second when the results of more than two groups were studied simultaneously.

The hypotheses proposed for each of the comparative analyses of the variables were:

Null hypothesis (H0): the results obtained by students in each group are the similar.
Alternative hypothesis (H1): the results obtained by the students in each group are different.

Again, the significance level chosen for decision-making was *p* = 0.05.

### Ethics Statement

In order to perform this research, written permission was first obtained from the Alonso de Avellaneda Vocational Education and Training School faculty board (in Spanish, “Equipo Directivo”), which serves as our ethics committee. The faculty board authorized and oversaw the proceedings used in data collection and processing in addition to approval of all aspects of the study protocol.

Pursuant to Organic Law 15/1999’s article 5 of December 13 regarding Protection of Data of a Personal Nature, when students applied for registration, they (or their parents/guardians if minors) provided written authorization for their personal data to be collected. Furthermore, article 4 of the aforementioned law indicates that such data can be processed at a future date for historical, statistical, or scientific purposes. Therefore, the faculty board (ethics committee) does not consider it necessary to request additional authorization to perform this statistical study.

In addition, there is no sensitive information in the data utilized and all data has been collected in an anonymous manner. An identification number was assigned to each student rather than a name to insure anonymity.

Surveys carried out with students via online distance modes (all adults) were conducted using an online platform in a voluntary and fully anonymous manner. Students gave their written consent at the time the online survey was conducted so its results could be analyzed.

Information collected was not in any way used in the process of grading or evaluating students.

Our goal is to use this study’s results to promote our educational center, IES Alonso de Avellaneda, professional training in general, and specifically and especially to further online distance teaching and the use of virtual learning environments (VLEs) in official vocational education and training in Spain.

## Results and Discussion

**Figure 1:**
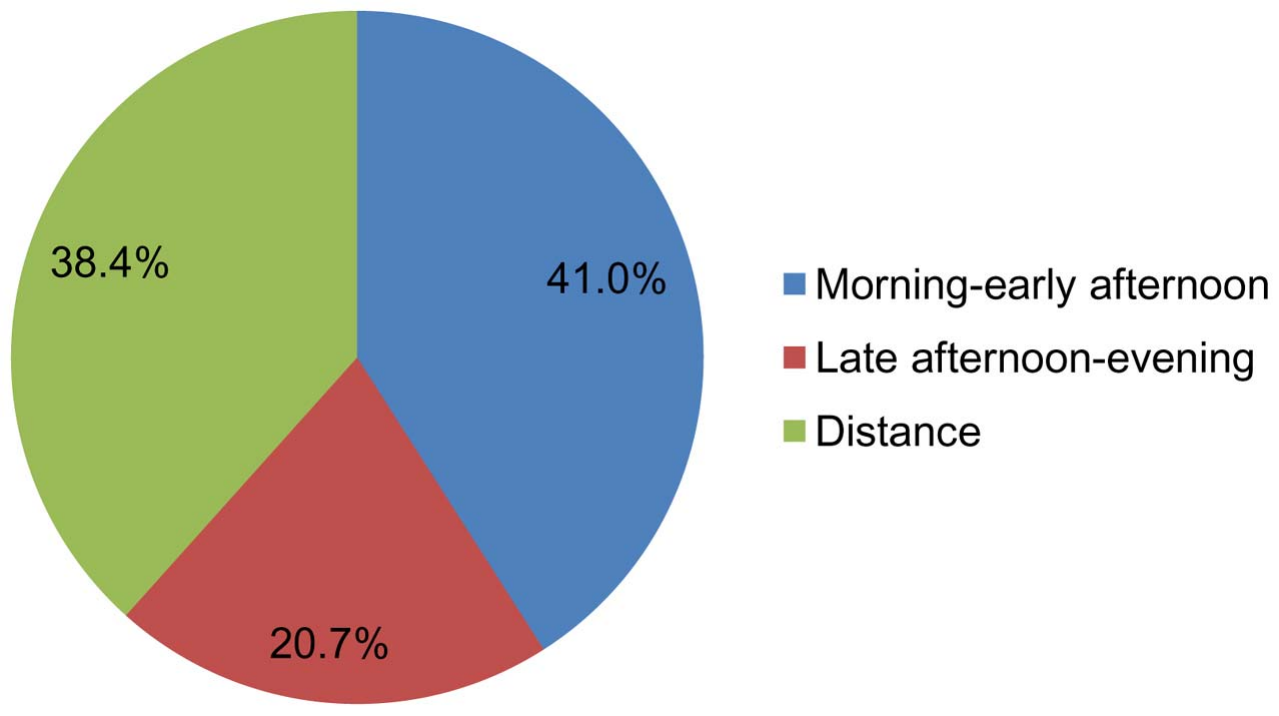
Distribution of students by morning-early afternoon, late afternoon-evening or distance mode.

The 1,133 students enrolled in the educational program during the eleven academic years studied were distributed in the following manner: 464 (41%) in the morning-early afternoon class, 234 (20.7%) in the late afternoon-evening class and 435 (38.4%) in the distance mode, as can be seen in Figure 1.

The age variable contained values ranging between 15 and 58 years, with an average of 23.5 years and a standard deviation of 7.4 years. The educational program thus served students of very different ages covering a range of 43 years. Analyzing the age variable for each of the modalities and class times, the results indicate that for the morning-early afternoon classes, the mean for the age variable is 18 years with an interquartile range of 2 years; for the late afternoon-evening classes the average is 21 years with an interquartile range is 5.5 years, and in distance mode the average is 25 years with an interquartile range 10 years.

**Figure 2:**
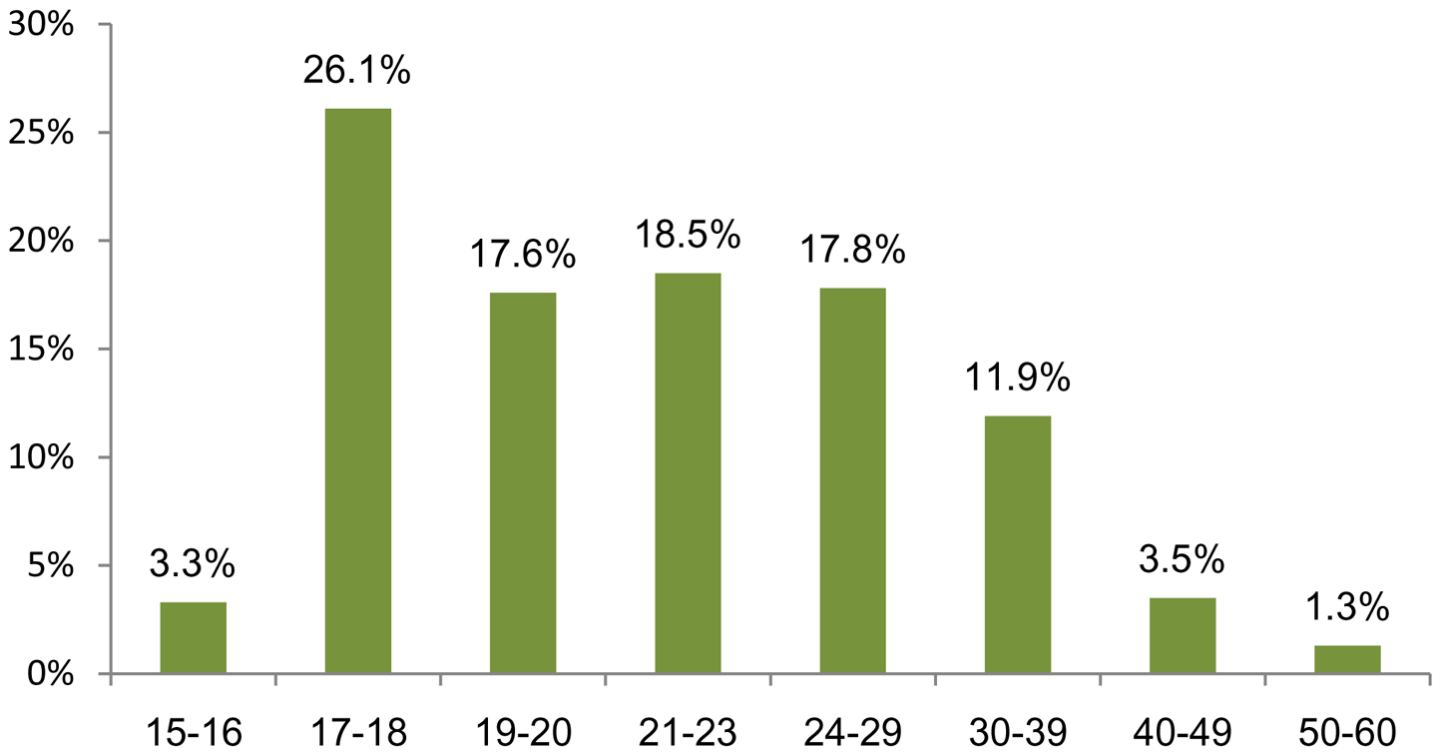
Distribution of students by age grouping.

The youngest students typically enroll in the program’s morning-early afternoon classes. Many of the students in the late afternoon-evening classes work during the morning-early afternoon, and those enrolled through the distance mode are often unable to attend either classroom time. As seen in Figure 2, the majority of students (80%) are aged 17–29 years, although there is a significant group of older students (11.9%) aged 30 - 39.

When performed for the age variable, the Kruskal-Wallis test confirms that this variable’s median value is different for each of the class times and for distance mode, with a significance level of 1%.

With a broad and flexible offering, it is possible for most students to find a training program compatible with their other personal, professional, or family activities. In fact, the data analyzed indicate that the majority (85%) of students enrolled through distance mode are either working or looking for work, and enroll in the education program in order to obtain training and an official certification that will allow them to advance in the workplace or find a better job. The need for the morning-early afternoon and late afternoon-evening classroom sessions in addition to the distance mode is also validated, since each of these responds to different types of needs. The teaching program as a whole is thus adapted to the individual circumstances of all students.

**Figure 3:**
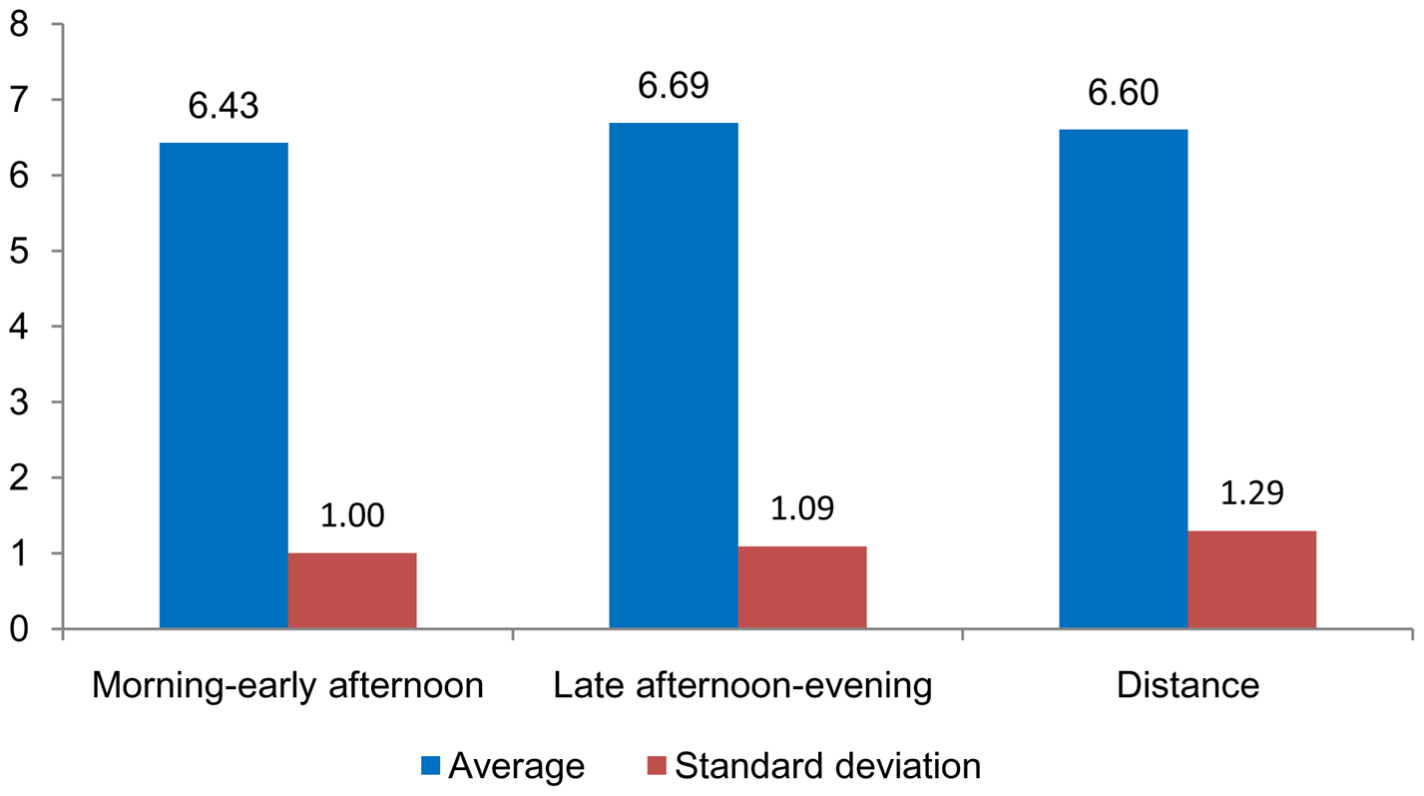
Average grade awarded for morning-early afternoon, late afternoon-evening and distance mode with standard deviation.

In terms of the results when analyzing the average grade variable (with Spain’s 10-point grading scale), the average value for the morning-early afternoon classes is 6.43 points (standard deviation = 1.0 point), the average for the late afternoon-evening classes is 6.69 points (standard deviation = 1.1 point), and for distance mode the average is 6.6 points (standard deviation = 1.3 points), (Figure 3).

The differences in the average grades awarded to students enrolled in the morning-early afternoon or late afternoon-evening classes or in distance mode are small. However, the statistical analysis does show that the average grade for the late afternoon-evening classes is significantly higher (*p = *0.05). Throughout the period studied, using a significance level of *p* = 0.05 there is no significant evidence that the average grades for students in the morning-early afternoon classes or distance mode differ significantly from each other.

**Figure 4:**
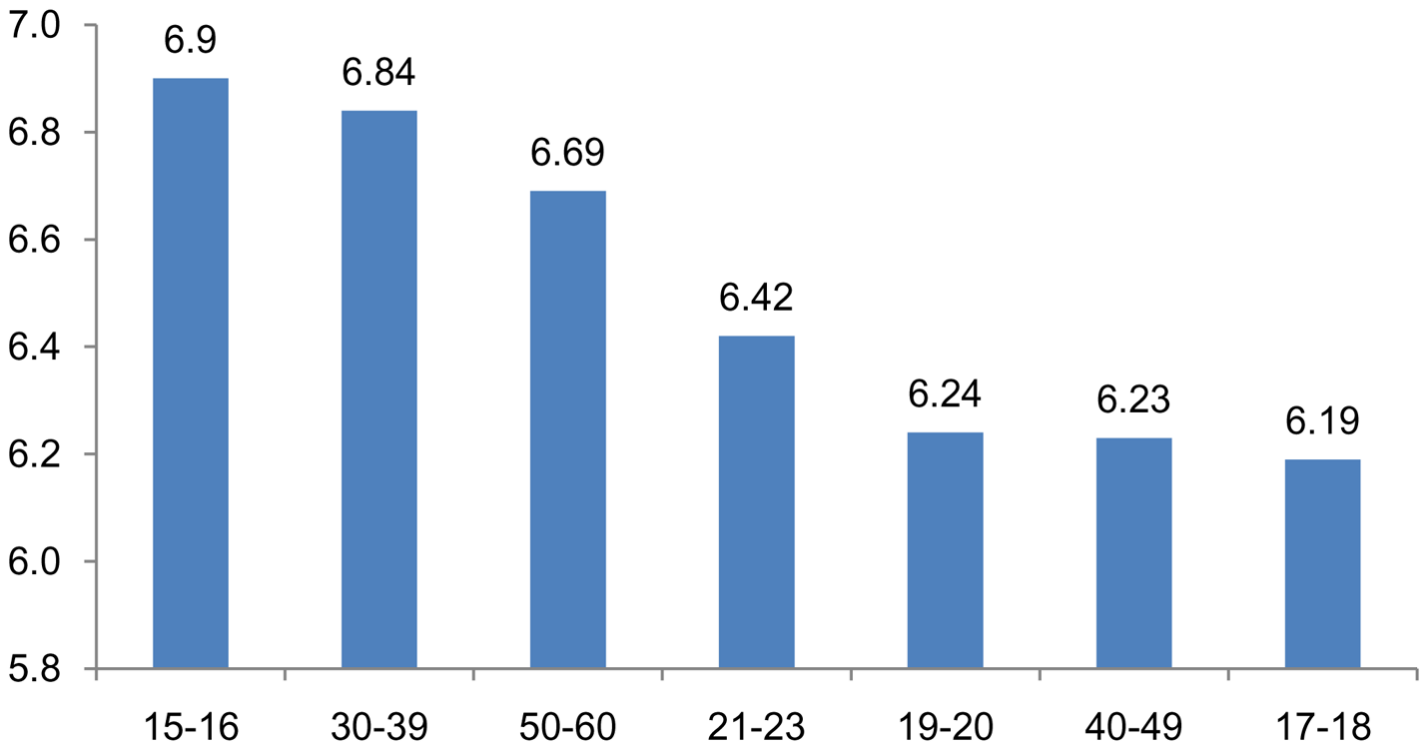
Average grades by age group.

When average grades are analyzed by age group, other differences can be observed, as seen in Figure 4.

Students with the highest average grade are those from 24–29 years old, followed by the students aged 15–16. The lowest grades were seen for the 17–18 year-old students. Students older than age 20 begin the educational program with high levels of motivation and this influences their results, despite the fact that the great majority of them are also working. The 15–16 year old group also showed high marks, and these are the students who were not required to repeat any school years during their first phase of high school (Compulsory Secondary Education) and who decided to enroll in a vocational program rather than continue along the more academic track of the second-phase high-school curriculum (called *Bachillerato*). The group of students between 17 and 18 years old is typically made up of students who repeated at least one school year during the mandatory-attendance first phase of high school.

In terms of the data related to advancement, in the morning-early afternoon class group 50.3% were promoted, for the late afternoon-evening group 58.5%, and for distance mode 32.4%. Here again the late afternoon-evening classroom students show the best results while the advancement rate for the distance mode students is substantially lower than those seen in the two classroom-learning groups. However, the advancement percentage for the students who follow the program correctly showed a rate of 51.6%, a number similar to that seen for the morning-early afternoon classroom group. The online distance mode students also showed better results than the morning-early afternoon and/or late afternoon-evening classroom students for other variables, such as average grades for certain individual course years or average grades for certain subjects presented during the vocational program.

Analysis of the results for the withdrawal variable clearly show that the youngest students are the ones least likely to drop out of the program. These students typically are not working and are studying Administrative Management as their primary activity. They tend to begin the program immediately after completing their mandatory first phase of high school and therefore have stayed continuously within the educational system. When evaluating the hypotheses in relation to the withdrawal variable, there is statistical evidence that the attrition rate differs for the three main groups, with a significance level of 1%. Specifically by percentages, the withdrawal rate for the morning-early afternoon, late afternoon-evening, and distance groups are 8.8%, 9.4%, and 53.2%, respectively. Such high percentages of withdrawals is one of the most important problems still facing distance learning programs and high rates such as these are also seen for university-level studies [31].

**Figure 5:**
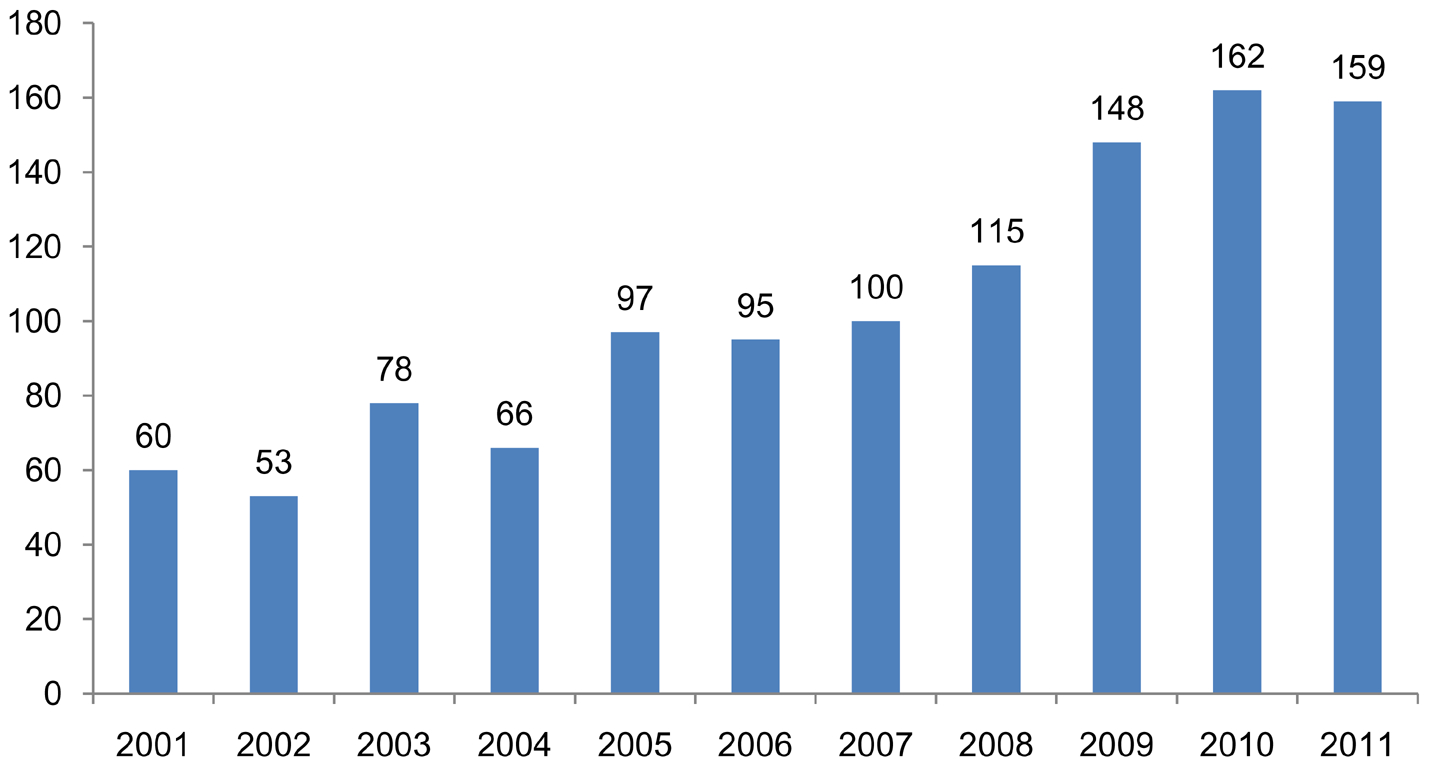
Number of students enrolled during each academic year.

The enrollment number data show that the overall number of students in the program has increased notably, especially after the distance mode was implemented. During the 2000–2001 academic year there were 60 students enrolled in the vocational program, while during the 2010–2011 year the number of students was 159, (Figure 5).

The contribution that late afternoon-evening classes and online distance learning options made to vocational training programs is very important. Changes in the employment structures seen in Spain in recent years, especially the loss of construction sector jobs, have required many workers to expand their range of training and skills to look for work in new industries. The training program being discussed here offers vocational training to students immediately following their mandatory high school phase as well as to already active workers who want to improve their degree of formal qualifications. The important role played by online distance education in the vocational training model can thus be confirmed, since along with classroom learning it is helping to supply the increasing demand for vocational education and training now existing in Spain.

### Evaluation of the Virtual Platform

An important part of the process for establishing and improving online education is proper evaluation of the system. The quality of the Moodle-based courses implemented at the Alonso de Avellaneda Vocational Education and Training School has been evaluated using the “Quality on the Line” model [32], which was created to evaluate distance learning systems offered via the Internet. This model consists of a series of questions designed to evaluate the structure and presentation of the classes, the support offered to students and instructors, the teaching-learning process, and the student evaluation procedures. The results produced are good, although there is a need for improvement of the aspects related to the support received by the instructors who provide online distance vocational learning. Up until now, there has been no system of recognition to provide incentives for teachers, which would contribute to improvement of quality and ensure the continuity of this mode of vocational training.

The survey carried out with students enrolled in the online distance learning mode followed the COLLES method (Constructivist On-Line Learning Environment Survey) [33], and provided evidence for the fundamental and value-adding role of the platform in the teaching and learning process. Students that took the survey through the virtual platform did so in a voluntary manner at the end of two academic course cycles. 42 students participated. The student response results for each question asked in the COLLES survey can be seen in Table 1.

**Table 1:** COLLES survey summary table.

		Almost always (5)		Often (4)		Sometimes (3)		Seldom (2)		Almost Never (1)		Statistics		
		Abs.Freq.	Rel.Freq.	Abs.Freq.	Rel.Freq.	Abs.Freq.	Rel.Freq.	Abs.Freq.	Rel.Freq.	Abs.Freq.	Rel.Freq.	Mean	Mode	Var.
Relevance	My learning focuses on issues that interest me	52%	22	48%	20	0%	0	0%	0	0%	0	4.5	5	0.3
	What I learn is important for my professional practice	48%	20	43%	18	10%	4	0%	0	0%	0	4.4	5	0.4
	I learn how to improve my professional practice	62%	26	29%	12	10%	4	0%	0	0%	0	4.5	5	0.5
	What I learn connects well with my professional practice	43%	18	43%	18	10%	4	5%	2	0%	0	4.2	4	0.7
Reflection	I think critically about how I learn	19%	8	48%	20	24%	10	10%	4	0%	0	3.8	4	0.8
	I think critically about my own ideas	29%	12	43%	18	10%	4	10%	4	10%	4	3.7	4	1.6
	I think critically about other students’ ideas	5%	2	24%	10	29%	12	19%	8	24%	10	2.7	3	1.5
	I think critically about ideas in the readings	24%	10	33%	14	33%	14	5%	2	5%	2	3.7	4	1.1
Interactivity	I explain my ideas to other students	14%	6	5%	2	52%	22	10%	4	19%	8	2.9	3	1.5
	I ask other students to explain their ideas	14%	6	10%	4	48%	20	0	8	0	4	3.0	3	1.3
	Other students ask me to explain my ideas	5%	2	10%	4	38%	16	29%	12	19%	8	2.5	3	1.1
	Other students respond to my ideas	14%	6	5%	2	48%	20	14%	6	19%	8	2.8	3	1.5
Tutor support	The tutor stimulates my thinking	14%	6	29%	12	48%	20	10%	4	0%	0	3.5	3	0.7
	The tutor encourages me to participate	43%	18	24%	10	29%	12	5%	2	0%	0	4.0	5	0.9
	The tutor models good discourse	38%	16	14%	6	38%	16	5%	2	5%	2	3.8	3	1.4
	The tutor models critical self-reflection	24%	10	29%	12	33%	14	10%	4	5%	2	3.6	3	1.2
Peer support	Other students encourage my participation	14%	6	24%	10	24%	10	29%	12	10%	4	3.0	2	2.0
	Other students praise my contribution	5%	2	5%	2	38%	16	33%	14	19%	8	2.4	3	1.0
	Other students value my contribution	10%	4	5%	2	43%	18	19%	8	24%	10	2.6	3	1.4
	Other students empathise with my struggle to learn	5%	2	14%	6	33%	14	24%	10	24%	10	2.5	3	1.3
Interpretation	I make good sense of other students’ messages	43%	18	33%	14	24%	10	0%	0	0%	0	4.2	5	0.6
	Other students make good sense of my messages	33%	14	33%	14	33%	14	0%	0	0%	0	4.0	5	0.7
	I make good sense of the tutor’s messages	62%	26	19%	8	19%	8	0%	0	0%	0	4.4	5	0.6
	The tutor makes good sense of my messages	57%	24	19%	8	19%	8	5%	2	0%	0	4.3	5	0.9

Abs. Freq.: absolute frequency, Rel. Freq.: relative frequency, Var.: variance.

The questions posed to the students could be answered using one of the following options: almost always; often; sometimes; seldom; or almost never. The main subjects covered by the survey were:

Relevance.
Reflection.
Interactivity.
Tutor support.
Peer support
Interpretation.

The results showed that for the majority (86%) of the Alonso de Avellaneda Vocational Education and Training School’s distance-mode students, the studies had relevance almost always or often for their vocational practice. Many of these students were already working in the administrative sector at one level or another, which helped give the contents of the training program direct relevance to their workplace activities.

Reflective thinking (reflection) is another important point for any educational program. Here the results indicated that most students (67%) did engage in critical thinking about their own learning process almost always or often while the remaining 33% do it sometimes or seldom. Regarding critical thinking about their own ideas, 72% of students responded often or almost always while 19% sometimes or seldom. Regarding critical thinking about the ideas of fellow students, results are more divided between those who do it normally and those who do it much less frequently given that 53% answered sometimes or often while 43% responded seldom or almost never.

Their ability to share ideas with their classmates is made possible by the collaborative approach and environment created within the context of the program’s virtual classroom. In terms of interactivity, responses were more centered on sometimes than almost always or often. Results indicate that a significant percentage of students (48%) interact by asking questions or explanations from their peers and a significant number (38%) perceive that their peers ask questions sometimes. Although the students regularly visit the virtual classroom’s online forums, they less frequently post their own questions there. However, some questions can be answered simply by reading the previously posted questions and answers. On the other hand, the results in this section are also logical since the instructors are typically the ones to provide the answers to questions.

The responses from the students clearly show that the tutor is very present during the entire teaching-learning process. The majority of the students (67%) gave the response almost always or often for the tutor encourages me to participate. A significant percentage (43%) is of the opinion that the tutor often or sometimes stimulates reflective thinking and another 48% feels that this motivation is done sometimes. In addition, the tutor moderates and provides examples according to the opinion of 90% of students almost always, often, or sometimes. The tutor support is therefore of key importance, including for contact with the students through the virtual classroom, and the messages of encouragement such as those provided along with responses to theoretical questions are helpful.

The need to study alone is another aspect of distance learning that can make continuing participation more difficult. Most students (61%) responded with sometimes or seldom to questions related to the feeling of being supported by peers. However, although the results reinforce the idea that support from the tutor is more present than support from other students, it is the sum of the two types of support that is most important.

Regarding communication through the virtual classroom (interpretation), 81% of students responded that they understood the tutor’s messages well almost always or often. In addition, the majority of students (76%) think that the tutor understands messages he/she receives almost always or often. Questions related to communication with peers in the virtual classroom show that 73% of students always or almost always understand messages from other students. In response to the question: other students understand my messages well, 66% of students responded almost always or often. This section’s results confirm that the flow of communication is regular and takes place in a collaborative manner between participants sharing the virtual learning space.

Other questions asked of the students include whether they were working or looking for work, as well as the reason for their decision to enroll in the vocational program. These results showed that 95% of the students were working or looking for work in an active manner, and that the distance modality gave them the flexibility they needed in terms of scheduling. With respect to the reasons that led students to enroll in the Administrative Management program, the results showed that 60% wanted to obtain the certification in order to make a change away from their current job possible. Another 25% needed the qualification to improve their employment position, and another 5% in order to retain their current job. The remaining 10% enrolled in the vocational program for other reasons.

During the academic years since the program began to be offered via the virtual platform, students of varying ages, professional backgrounds, and educational levels have participated in the vocational training experience with a sense of closeness to their instructors and classmates. The feeling of physical distance has been reduced, and the messages of encouragement from the participants, the advice and orientation offered, the ability to receive responses to questions as soon as they arose, and the feeling of belonging to a group with a common set of goals have helped many to attain the skills and formal qualifications they needed.

## Conclusions

In Spain, the drastic contraction seen in the construction sector in recent years, in combination with changes in the production structure and increasing demand for educated workers in emerging sectors, has led to a notable increase in demand for professional training. Given this scenario, the role of vocational education and training in general and specifically its offering in online distance mode has become fundamental, since it allows flexible scheduling in terms of time and place for persons who would otherwise not have the opportunity to acquire new skills because of their existing personal and/or employment circumstances. The results of this study confirm the validity of the online distance learning mode as a means of responding to the educational needs of students with widely varying ages, as well as this mode’s ability to be adapted to the needs of official vocational training programs.

The data have also demonstrated that the online distance mode is comparable with the results seen for the morning-early afternoon and late afternoon-evening classroom groups, although work must continue on finding ways to reduce the dropout rate for online students. Educational opportunities offered at International Standard Classification of Education (ISCED 1997) level 3B must also be strengthened (Mid-Level Vocational Programs) so that the youngest students as well as active workers can complete or expand their education. Online distance education will continue to be a key factor in this process.

## Supporting Information

Table S1 Dataset.
(DOCX)
