# Supplementary material for: A Comparative Study of Classroom and Online Distance Modes of Official Vocational Education and Training
Source: PLoS One. 2014 May 1;9(5):e96052. doi: 10.1371/journal.pone.0096052 (PMC4006865; doi:10.1371/journal.pone.0096052)
Supplement: Table S1 — Dataset. (DOCX) [file pone.0096052.s001.docx]

Table S1. Dataset.

| **ID** | **S1** | **S2** | **S3** | **S4** | **S5** | **S6** | **S7** | **S8** | **Year** | **Classes** | **Group** | **Gender** | **Age** |
| --- | --- | --- | --- | --- | --- | --- | --- | --- | --- | --- | --- | --- | --- |
| 1133 | NE | NE | NE | NE | NE | NE | NE | NE | 2011 | D | A | Female | 32 |
| 1132 | 5 | 5 | 3 | NE | NE | NE | 3 | NE | 2011 | D | A | Female | 20 |
| 1131 | 2 | NE | NE | NE | NE | NE | 2 | NE | 2011 | D | A | Male | 26 |
| 1130 | NE | NE | NE | NE | NE | NE | NE | NE | 2011 | D | A | Male | 42 |
| 1129 | NE | NE | NE | NE | NE | NE | NE | NE | 2011 | D | A | Female | 41 |
| 1128 | 5 | W | 5 | 5 | 5 | 5 | W | 5 | 2011 | D | A | Female | 24 |
| 1127 | NE | NE | NE | NE | NE | NE | NE | NE | 2011 | D | A | Male | 30 |
| 1126 | 5 | 5 | NE | NE | NE | NE | NE | 5 | 2011 | D | A | Female | 25 |
| 1125 | 5 | 5 | 5 | NE | 5 | 5 | 2 | 5 | 2011 | D | A | Female | 24 |
| 1124 | NE | NE | NE | NE | NE | NE | NE | NE | 2011 | D | A | Female | 25 |
| 1123 | NE | NE | NE | W | W | W | NE | W | 2011 | D | A | Female | 35 |
| 1122 | 5 | 5 | 5 | 5 | 5 | 5 | 5 | 5 | 2011 | D | A | Female | 25 |
| 1121 | 5 | 5 | 5 | 5 | 5 | 5 | 10 | 5 | 2011 | D | A | Female | 44 |
| 1120 | 5 | 5 | 5 | W | 5 | 5 | W | 5 | 2011 | D | A | Female | 53 |
| 1119 | 5 | NE | NE | NE | 5 | NE | NE | NE | 2011 | D | A | Female | 21 |
| 1118 | NE | 5 | 5 | 5 | 5 | NE | NE | NE | 2011 | D | A | Female | 23 |
| 1117 | 5 | 7 | 5 | 6 | 5 | 5 | 3 | 5 | 2011 | D | A | Female | 23 |
| 1116 | NE | NE | NE | NE | NE | NE | NE | NE | 2011 | D | A | Female | 22 |
| 1115 | NE | 5 | 2 | NE | 3 | NE | 3 | NE | 2011 | D | A | Male | 24 |
| 1114 | NE | NE | NE | NE | NE | NE | NE | NE | 2011 | D | A | Male | 35 |
| 1113 | NE | NE | NE | NE | NE | NE | NE | NE | 2011 | D | A | Female | 33 |
| 1112 | NE | NE | NE | NE | NE | NE | NE | NE | 2011 | D | A | Female | 29 |
| 1111 | 5 | 5 | NE | 5 | NE | NE | 5 | 5 | 2011 | D | A | Male | 27 |
| 1110 | NE | NE | NE | NE | NE | NE | NE | NE | 2011 | D | A | Female | 27 |
| 1109 | W | W | W | W | W | NE | W | W | 2011 | D | A | Male | 28 |
| 1108 | NE | NE | NE | NE | NE | NE | 5 | NE | 2011 | D | A | Female | 30 |
| 1107 | NE | NE | NE | NE | NE | NE | NE | NE | 2011 | D | A | Male | 27 |
| 1106 | NE | 5 | 5 | NE | NE | 5 | NE | 5 | 2011 | D | A | Male | 25 |
| 1105 | NE | NE | 6 | 3 | 5 | 5 | 3 | 5 | 2011 | D | A | Female | 30 |
| 1104 | 5 | 5 | 5 | 5 | 5 | 5 | 5 | 5 | 2011 | D | A | Female | 39 |
| 1103 | 5 | 5 | NE | NE | NE | 5 | 5 | NE | 2011 | D | A | Female | 30 |
| 1102 | 5 | 8 | 5 | 5 | 5 | 5 | 5 | 5 | 2011 | D | A | Female | 23 |
| 1101 | W | W | W | W | W | W | W | W | 2011 | D | A | Female | 26 |
| 1100 | NE | NE | NE | NE | NE | NE | NE | 5 | 2011 | D | A | Female | 23 |
| 1099 | NE | NE | NE | NE | NE | NE | 5 | 5 | 2011 | D | A | Male | 24 |
| 1098 | NE | NE | NE | NE | NE | NE | NE | NE | 2011 | D | A | Female | 26 |
| 1097 | NE | NE | NE | 5 | 5 | NE | 5 | 5 | 2011 | D | A | Female | 32 |
| 1096 | 5 | 3 | 5 | NE | 7 | 5 | NE | 4 | 2011 | D | A | Male | 29 |
| 1095 | NE | 7 | NE | NE | NE | NE | NE | NE | 2011 | D | A | Female | 31 |
| 1094 | 5 | 5 | 5 | 6 | 5 | 5 | 10 | 5 | 2011 | D | A | Female | 31 |
| 1093 | NE | NE | NE | NE | NE | NE | NE | NE | 2011 | D | A | Female | 52 |
| 1092 | NE | 3 | NE | NE | 5 | 5 | 5 | 5 | 2011 | D | A | Female | 30 |
| 1091 | NE | NE | NE | NE | NE | NE | NE | NE | 2011 | D | A | Female | 33 |
| 1090 | NE | NE | NE | NE | NE | NE | NE | NE | 2011 | D | A | Female | 35 |
| 1089 | 5 | NE | NE | 5 | 5 | NE | NE | 5 | 2011 | D | A | Female | 21 |
| 1088 | W | W | 5 | W | W | W | W | W | 2011 | D | A | Male | 37 |
| 1087 | NE | NE | NE | NE | NE | NE | NE | NE | 2011 | D | A | Female | 30 |
| 1086 | 7 | 5 | 5 | 5 | 5 | 5 | 9 | 5 | 2011 | D | A | Female | 20 |
| 1085 | 5 | 5 | 5 | 5 | 5 | 5 | 5 | 5 | 2011 | D | A | Female | 32 |
| 1084 | NE | NE | NE | NE | NE | NE | NE | NE | 2011 | D | A | Female | 20 |
| 1083 | 5 | 5 | 5 | 5 | 5 | 5 | 9 | 5 | 2011 | D | A | Female | 39 |
| 1082 | NE | 7 | NE | 5 | NE | 5 | NE | NE | 2011 | D | A | Female | 45 |
| 1081 | NE | 5 | 5 | NE | 5 | NE | NE | 2 | 2011 | D | A | Female | 22 |
| 1080 | 5 | 5 | 5 | NE | 5 | NE | 5 | 5 | 2011 | D | A | Female | 22 |
| 1079 | NE | NE | NE | NE | NE | NE | NE | NE | 2011 | D | A | Male | 36 |
| 1078 | 5 | 5 | NE | NE | NE | 5 | 5 | NE | 2011 | D | A | Female | 25 |
| 1077 | NE | NE | NE | NE | NE | NE | NE | NE | 2011 | D | A | Male | 46 |
| 1076 | 5 | 5 | 5 | 5 | 5 | 5 | 5 | 5 | 2011 | D | A | Male | 36 |
| 1075 | 5 | 5 | 5 | 5 | 5 | 6 | 10 | 5 | 2011 | D | A | Female | 37 |
| 1074 | 5 | 5 | 5 | 5 | 5 | 6 | 8 | 5 | 2011 | D | A | Female | 39 |
| 1073 | NE | 5 | NE | NE | NE | NE | NE | NE | 2011 | D | A | Male | 24 |
| 1072 | 5 | 8 | 5 | 5 | 5 | 5 | 5 | 5 | 2011 | D | A | Female | 30 |
| 1071 | 6 | 5 | 5 | 5 | 5 | 5 | 10 | 5 | 2011 | D | A | Male | 44 |
| 1070 | 5 | 8 | 7 | 7 | 7 | 5 | 5 | 5 | 2011 | D | A | Female | 26 |
| 1069 | NE | NE | NE | NE | NE | NE | NE | NE | 2011 | D | A | Female | 21 |
| 1068 | NE | 5 | 5 | NE | 5 | NE | 5 | 5 | 2011 | D | A | Female | 26 |
| 1067 | NE | NE | NE | NE | NE | NE | 5 | 5 | 2011 | D | A | Male | 23 |
| 1066 | NE | NE | NE | NE | NE | NE | NE | NE | 2011 | D | A | Male | 25 |
| 1065 | NE | NE | NE | NE | NE | NE | NE | NE | 2011 | D | A | Female | 25 |
| 1064 | NE | NE | NE | NE | NE | NE | NE | NE | 2011 | D | A | Male | 26 |
| 1063 | NE | NE | NE | W | W | W | NE | W | 2011 | D | A | Male | 36 |
| 1062 | W | W | W | W | W | W | W | 5 | 2011 | D | A | Female | 22 |
| 1061 | 5 | 5 | 5 | NE | 2 | NE | 5 | NE | 2011 | D | A | Female | 21 |
| 1060 | 5 | 6 | 3 | 3 | NE | NE | 4 | NE | 2011 | D | A | Female | 43 |
| 1059 | 9 | 9 | 5 | 5 | 5 | 5 | 10 | 5 | 2011 | D | A | Female | 37 |
| 1058 | 5 | 5 | 5 | 5 | 5 | NE | NE | 5 | 2011 | D | A | Female | 32 |
| 1057 | NE | NE | NE | NE | NE | NE | NE | NE | 2011 | D | A | Female | 28 |
| 1056 | NE | NE | NE | NE | NE | NE | NE | NE | 2011 | D | A | Female | 24 |
| 1055 | 6 | 4 | 4 | 5 | 5 | 5 | 9 | 6 | 2011 | E | A | Female | 20 |
| 1054 | NE | NE | NE | NE | NE | NE | NE | NE | 2011 | E | A | Female | 19 |
| 1053 | NE | NE | NE | W | NE | NE | W | NE | 2011 | E | A | Female | 55 |
| 1052 | 6 | 6 | 7 | 6 | 7 | 8 | 7 | 7 | 2011 | E | A | Female | 30 |
| 1051 | 8 | 6 | 6 | 6 | 6 | 6 | 10 | 9 | 2011 | E | A | Female | 37 |
| 1050 | NE | NE | NE | NE | NE | NE | NE | NE | 2011 | E | A | Female | 24 |
| 1049 | 5 | 4 | 4 | W | 4 | 4 | 4 | 5 | 2011 | E | A | Male | 28 |
| 1048 | NE | NE | NE | NE | NE | NE | NE | NE | 2011 | E | A | Female | 54 |
| 1047 | NE | NE | NE | NE | NE | NE | NE | NE | 2011 | E | A | Male | 19 |
| 1046 | 6 | 6 | 6 | 6 | 6 | 6 | 9 | 8 | 2011 | E | A | Female | 21 |
| 1045 | NE | 2 | 2 | 2 | NE | 3 | 3 | NE | 2011 | E | A | Female | 19 |
| 1044 | 8 | 6 | 6 | 7 | 7 | 7 | 10 | 8 | 2011 | E | A | Female | 52 |
| 1043 | W | W | W | W | W | W | W | W | 2011 | E | A | Female | 21 |
| 1042 | 6 | 5 | 5 | 5 | 5 | 5 | 8 | 7 | 2011 | E | A | Female | 24 |
| 1041 | W | W | W | W | W | W | W | W | 2011 | E | A | Male | 22 |
| 1040 | 6 | 5 | 6 | 6 | 5 | 5 | 10 | 7 | 2011 | E | A | Male | 17 |
| 1039 | 9 | 9 | 9 | 9 | 10 | 8 | 10 | 10 | 2011 | E | A | Female | 24 |
| 1038 | W | W | W | 8 | W | W | 9 | W | 2011 | E | A | Female | 30 |
| 1037 | 5 | 5 | 5 | 5 | 4 | 5 | 5 | 5 | 2011 | E | A | Female | 24 |
| 1036 | 5 | 3 | 3 | W | 3 | W | 4 | 6 | 2011 | E | A | Male | 26 |
| 1035 | 10 | 9 | 8 | 10 | 9 | 9 | 10 | 10 | 2011 | E | A | Male | 28 |
| 1034 | NE | NE | NE | NE | NE | NE | NE | NE | 2011 | E | A | Female | 18 |
| 1033 | 6 | 7 | 8 | 8 | 8 | 7 | 8 | 8 | 2011 | E | A | Male | 35 |
| 1032 | NE | NE | NE | NE | NE | 2 | NE | NE | 2011 | E | A | Female | 20 |
| 1031 | 10 | 9 | 8 | 10 | 8 | 8 | 10 | 10 | 2011 | E | A | Female | 28 |
| 1030 | 6 | 7 | 7 | 8 | 7 | 7 | 9 | 8 | 2011 | E | A | Female | 19 |
| 1029 | W | W | W | W | W | W | W | W | 2011 | E | A | Male | 27 |
| 1028 | 5 | NE | 5 | NE | 7 | 2 | 7 | 2 | 2011 | M | B | Female | 19 |
| 1027 | 4 | 3 | 2 | NE | 4 | 2 | 4 | 2 | 2011 | M | B | Male | 19 |
| 1026 | NE | NE | NE | NE | NE | NE | NE | NE | 2011 | M | B | Male | 19 |
| 1025 | NE | NE | NE | NE | NE | 2 | NE | NE | 2011 | M | B | Male | 19 |
| 1024 | 7 | 10 | 8 | 10 | 9 | 8 | 8 | 6 | 2011 | M | B | Female | 24 |
| 1023 | 5 | 8 | 6 | NE | 9 | 3 | 7 | 5 | 2011 | M | B | Female | 20 |
| 1022 | NE | NE | 2 | NE | NE | NE | NE | NE | 2011 | M | B | Female | 20 |
| 1021 | 6 | 5 | 4 | 2 | 5 | 5 | 7 | 5 | 2011 | M | B | Female | 18 |
| 1020 | 7 | 6 | 7 | 5 | 9 | 6 | 7 | 6 | 2011 | M | B | Male | 18 |
| 1019 | 6 | 8 | 8 | 6 | 6 | 6 | 7 | 6 | 2011 | M | B | Female | 20 |
| 1018 | 3 | 3 | 4 | NE | 2 | 2 | 4 | 4 | 2011 | M | B | Male | 18 |
| 1017 | 8 | 7 | 8 | 9 | 9 | 8 | 9 | 7 | 2011 | M | B | Male | 20 |
| 1016 | 3 | 3 | 2 | NE | 4 | 2 | 2 | NE | 2011 | M | B | Female | 18 |
| 1015 | NE | NE | NE | NE | NE | NE | NE | NE | 2011 | M | B | Male | 18 |
| 1014 | 6 | 7 | 6 | 6 | 7 | 5 | 6 | 5 | 2011 | M | B | Male | 22 |
| 1013 | 7 | 7 | 6 | 9 | 7 | 6 | 7 | 6 | 2011 | M | B | Male | 19 |
| 1012 | 5 | 5 | 5 | 5 | 6 | 3 | 5 | 3 | 2011 | M | B | Female | 18 |
| 1011 | 8 | 5 | 8 | 8 | 7 | 6 | 8 | 6 | 2011 | M | B | Female | 22 |
| 1010 | NE | NE | NE | NE | NE | NE | NE | NE | 2011 | M | B | Female | 20 |
| 1009 | 6 | 5 | 6 | 7 | 5 | 5 | 6 | 5 | 2011 | M | B | Male | 20 |
| 1008 | 7 | 5 | 6 | 5 | 5 | 6 | 6 | 6 | 2011 | M | B | Female | 19 |
| 1007 | 8 | 5 | 6 | 8 | 6 | 6 | 8 | 6 | 2011 | M | B | Female | 22 |
| 1006 | NE | NE | NE | NE | NE | NE | NE | NE | 2011 | M | B | Female | 18 |
| 1005 | 6 | 5 | 6 | NE | 5 | 5 | 5 | 5 | 2011 | M | B | Female | 18 |
| 1004 | 8 | 8 | 8 | 10 | 9 | 8 | 7 | 7 | 2011 | M | B | Male | 19 |
| 1003 | 5 | 6 | 5 | 8 | 5 | 5 | 6 | 5 | 2011 | M | B | Male | 20 |
| 1002 | NE | NE | NE | NE | NE | NE | NE | NE | 2011 | M | A | Male | 23 |
| 1001 | 3 | 3 | 3 | 2 | 3 | 2 | 6 | 2 | 2011 | M | A | Male | 19 |
| 1000 | 5 | 5 | 5 | NE | 7 | NE | 6 | 5 | 2011 | M | A | Female | 19 |
| 999 | NE | NE | NE | NE | NE | NE | NE | NE | 2011 | M | A | Female | 21 |
| 998 | 3 | 3 | 5 | 3 | 3 | 2 | 6 | 2 | 2011 | M | A | Male | 20 |
| 997 | 6 | 5 | 5 | 5 | 5 | 6 | 6 | 5 | 2011 | M | A | Male | 21 |
| 996 | 5 | 7 | 5 | 7 | 5 | NE | 7 | 5 | 2011 | M | A | Female | 20 |
| 995 | 8 | 7 | 8 | 9 | 5 | 7 | 7 | 6 | 2011 | M | A | Female | 22 |
| 994 | 7 | 7 | 6 | 5 | 7 | 7 | 6 | 5 | 2011 | M | A | Male | 19 |
| 993 | 5 | 6 | 5 | 5 | 6 | 3 | 6 | 5 | 2011 | M | A | Female | 19 |
| 992 | 5 | 5 | 5 | 3 | 5 | 5 | 5 | 2 | 2011 | M | A | Female | 20 |
| 991 | 5 | 5 | 5 | 9 | 5 | 7 | 5 | 5 | 2011 | M | A | Female | 23 |
| 990 | NE | NE | NE | NE | NE | NE | NE | NE | 2011 | M | A | Female | 22 |
| 989 | 3 | 3 | 3 | 5 | 5 | 3 | 2 | 2 | 2011 | M | A | Male | 21 |
| 988 | 3 | 5 | 3 | 5 | NE | NE | 6 | 3 | 2011 | M | A | Male | 19 |
| 987 | NE | NE | NE | NE | NE | NE | NE | NE | 2011 | M | A | Male | 18 |
| 986 | 5 | 6 | 5 | 6 | 6 | 3 | 7 | 5 | 2011 | M | A | Female | 18 |
| 985 | NE | NE | NE | NE | NE | NE | NE | NE | 2011 | M | A | Male | 18 |
| 984 | NE | NE | NE | NE | NE | NE | NE | NE | 2011 | M | A | Female | 19 |
| 983 | 5 | 5 | 8 | 8 | 5 | 5 | 7 | 5 | 2011 | M | A | Female | 22 |
| 982 | 6 | 5 | 7 | NE | 7 | 7 | 3 | 6 | 2011 | M | A | Female | 19 |
| 981 | 5 | 5 | 8 | 5 | 5 | 5 | 5 | 5 | 2011 | M | A | Female | 21 |
| 980 | 3 | 6 | 6 | 5 | 5 | 6 | 7 | 5 | 2011 | M | A | Female | 20 |
| 979 | 5 | 8 | 5 | 7 | 5 | NE | 5 | 5 | 2011 | M | A | Female | 19 |
| 978 | 10 | 7 | 8 | 7 | 9 | 7 | 7 | 6 | 2011 | M | A | Female | 28 |
| 977 | 6 | 6 | 5 | 8 | 5 | 6 | 7 | 5 | 2011 | M | A | Female | 23 |
| 976 | 2 | 3 | 3 | NE | NE | 2 | NE | 2 | 2011 | M | A | Female | 20 |
| 975 | NE | NE | NE | NE | NE | NE | NE | NE | 2011 | M | A | Male | 18 |
| 974 | 5 | 5 | 5 | NE | NE | 3 | 5 | NE | 2010 | D | A | Female | 32 |
| 973 | NE | NE | NE | NE | NE | NE | NE | NE | 2010 | D | A | Female | 19 |
| 972 | 5 | 5 | NE | 5 | 5 | 6 | 5 | 5 | 2010 | D | A | Female | 24 |
| 971 | NE | NE | NE | NE | NE | NE | NE | NE | 2010 | D | A | Female | 31 |
| 970 | NE | NE | NE | NE | NE | NE | NE | NE | 2010 | D | A | Female | 23 |
| 969 | NE | NE | 5 | 5 | 5 | NE | 5 | NE | 2010 | D | A | Female | 20 |
| 968 | 8 | 10 | 7 | 6 | 8 | 6 | 10 | 7 | 2010 | D | A | Female | 33 |
| 967 | NE | NE | NE | NE | NE | NE | NE | NE | 2010 | D | A | Female | 26 |
| 966 | 7 | 6 | 4 | NE | 4 | 4 | 4 | 6 | 2010 | D | A | Female | 24 |
| 965 | 5 | NE | 5 | NE | NE | NE | 5 | 5 | 2010 | D | A | Female | 27 |
| 964 | 5 | 5 | NE | 5 | NE | NE | 5 | 5 | 2010 | D | A | Female | 47 |
| 963 | 8 | 9 | 7 | NE | 6 | 7 | 3 | 7 | 2010 | D | A | Female | 23 |
| 962 | NE | NE | NE | NE | NE | NE | NE | NE | 2010 | D | A | Female | 23 |
| 961 | NE | NE | NE | NE | NE | NE | NE | NE | 2010 | D | A | Female | 23 |
| 960 | 5 | 5 | 5 | 5 | 5 | W | 5 | 5 | 2010 | D | A | Female | 24 |
| 959 | NE | NE | NE | W | W | NE | NE | NE | 2010 | D | A | Female | 19 |
| 958 | 7 | 7 | 6 | W | 5 | 7 | W | 7 | 2010 | D | A | Female | 52 |
| 957 | NE | NE | NE | NE | NE | 2 | NE | 5 | 2010 | D | A | Female | 23 |
| 956 | NE | NE | NE | NE | NE | NE | NE | NE | 2010 | D | A | Female | 19 |
| 955 | 4 | 4 | 3 | NE | NE | 5 | 4 | 5 | 2010 | D | A | Female | 23 |
| 954 | NE | NE | NE | NE | NE | NE | NE | NE | 2010 | D | A | Female | 29 |
| 953 | NE | NE | NE | NE | NE | NE | NE | NE | 2010 | D | A | Female | 21 |
| 952 | NE | NE | NE | NE | NE | NE | NE | NE | 2010 | D | A | Female | 36 |
| 951 | NE | NE | 5 | NE | 5 | NE | 5 | 5 | 2010 | D | A | Female | 25 |
| 950 | NE | NE | NE | NE | NE | 2 | NE | NE | 2010 | D | A | Female | 28 |
| 949 | 5 | 5 | NE | 5 | NE | NE | 5 | 5 | 2010 | D | A | Female | 26 |
| 948 | NE | NE | 2 | NE | NE | NE | 6 | NE | 2010 | D | A | Female | 29 |
| 947 | W | W | W | W | W | W | W | W | 2010 | D | A | Female | 60 |
| 946 | 10 | 9 | 7 | W | W | W | 8 | 6 | 2010 | D | A | Female | 38 |
| 945 | NE | NE | NE | NE | NE | NE | NE | NE | 2010 | D | A | Female | 25 |
| 944 | 8 | 8 | W | W | W | 6 | 9 | W | 2010 | D | A | Female | 29 |
| 943 | NE | NE | NE | NE | NE | NE | NE | NE | 2010 | D | A | Female | 23 |
| 942 | NE | NE | NE | NE | NE | NE | NE | NE | 2010 | D | A | Female | 24 |
| 941 | NE | NE | NE | NE | NE | NE | NE | NE | 2010 | D | A | Female | 21 |
| 940 | 8 | 8 | 7 | 6 | 8 | 7 | 8 | 7 | 2010 | D | A | Female | 25 |
| 939 | 5 | 5 | 5 | 5 | 9 | 9 | 5 | 5 | 2010 | D | A | Female | 30 |
| 938 | 5 | 5 | 5 | 5 | 6 | 5 | 5 | 5 | 2010 | D | A | Female | 26 |
| 937 | NE | 5 | NE | 5 | 5 | 5 | NE | NE | 2010 | D | A | Female | 21 |
| 936 | 9 | 8 | 8 | 7 | 8 | 6 | 10 | 6 | 2010 | D | A | Female | 32 |
| 935 | 8 | 10 | 8 | 6 | 7 | 7 | 10 | 7 | 2010 | D | A | Female | 35 |
| 934 | 5 | 5 | NE | NE | NE | 5 | 5 | 5 | 2010 | D | A | Female | 33 |
| 933 | NE | NE | NE | NE | NE | NE | NE | NE | 2010 | D | A | Female | 25 |
| 932 | NE | NE | NE | NE | NE | 3 | NE | NE | 2010 | D | A | Female | 24 |
| 931 | 8 | 8 | 8 | 8 | 7 | 8 | 8 | 6 | 2010 | D | A | Female | 26 |
| 930 | NE | NE | NE | NE | NE | NE | 4 | 5 | 2010 | D | A | Female | 21 |
| 929 | W | W | W | W | W | W | W | W | 2010 | D | A | Female | 29 |
| 928 | NE | NE | NE | NE | NE | 2 | NE | NE | 2010 | D | A | Female | 19 |
| 927 | 5 | 5 | W | 5 | W | 5 | 5 | 5 | 2010 | D | A | Female | 31 |
| 926 | 5 | W | 5 | 5 | 5 | W | 5 | 5 | 2010 | D | A | Female | 24 |
| 925 | 5 | 10 | 5 | 10 | 10 | 10 | 10 | 5 | 2010 | D | A | Female | 45 |
| 924 | NE | NE | NE | NE | NE | NE | NE | NE | 2010 | D | A | Female | 28 |
| 923 | 5 | 5 | 5 | 6 | 5 | 5 | 8 | 5 | 2010 | D | A | Female | 20 |
| 922 | 8 | 8 | 6 | 5 | 7 | 6 | 8 | 7 | 2010 | D | A | Female | 40 |
| 921 | NE | NE | NE | NE | NE | NE | NE | 5 | 2010 | D | A | Female | 25 |
| 920 | NE | NE | 5 | NE | 5 | 5 | NE | 5 | 2010 | D | A | Female | 22 |
| 919 | NE | NE | NE | NE | NE | NE | NE | NE | 2010 | D | A | Female | 24 |
| 918 | NE | NE | NE | NE | NE | NE | NE | NE | 2010 | D | A | Female | 29 |
| 917 | NE | NE | NE | NE | NE | NE | NE | NE | 2010 | D | A | Female | 27 |
| 916 | NE | NE | NE | NE | NE | NE | NE | NE | 2010 | D | A | Female | 41 |
| 915 | NE | NE | NE | NE | NE | NE | NE | NE | 2010 | D | A | Female | 31 |
| 914 | 5 | 5 | NE | 5 | 5 | NE | 5 | NE | 2010 | D | A | Female | 40 |
| 913 | NE | NE | NE | NE | NE | 2 | NE | 5 | 2010 | D | A | Female | 22 |
| 912 | W | W | W | 5 | 5 | W | W | 5 | 2010 | D | A | Female | 51 |
| 911 | NE | NE | W | NE | W | W | NE | 5 | 2010 | D | A | Female | 21 |
| 910 | 10 | 10 | 9 | 9 | 10 | 10 | 9 | 7 | 2010 | D | A | Female | 40 |
| 909 | 8 | 5 | 2 | W | W | 5 | 5 | W | 2010 | D | A | Female | 24 |
| 908 | 5 | 5 | 8 | 5 | 5 | 5 | 5 | 8 | 2010 | D | A | Female | 35 |
| 907 | NE | NE | NE | NE | NE | 3 | NE | W | 2010 | D | A | Female | 29 |
| 906 | NE | NE | NE | NE | NE | NE | NE | NE | 2010 | D | A | Female | 30 |
| 905 | NE | NE | NE | NE | NE | NE | NE | NE | 2010 | D | A | Female | 21 |
| 904 | NE | W | 4 | W | W | W | NE | W | 2010 | D | A | Female | 37 |
| 903 | 5 | 5 | 7 | 7 | 7 | 5 | 9 | 5 | 2010 | D | A | Female | 24 |
| 902 | 8 | 10 | W | W | W | W | 9 | 5 | 2010 | D | A | Female | 36 |
| 901 | NE | NE | NE | NE | NE | NE | NE | NE | 2010 | D | A | Female | 39 |
| 900 | NE | NE | NE | NE | NE | NE | NE | NE | 2010 | D | A | Female | 43 |
| 899 | 8 | 9 | 5 | 6 | 5 | 4 | 4 | 5 | 2010 | D | A | Female | 31 |
| 898 | 5 | NE | NE | NE | NE | NE | 5 | 5 | 2010 | D | A | Female | 23 |
| 897 | 7 | W | 8 | 7 | W | W | 6 | W | 2010 | D | A | Female | 42 |
| 896 | NE | NE | NE | NE | NE | NE | NE | NE | 2010 | D | A | Female | 22 |
| 895 | 10 | 5 | 5 | 9 | 8 | 5 | 5 | 5 | 2010 | D | A | Female | 46 |
| 894 | NE | NE | NE | NE | NE | NE | NE | 5 | 2010 | D | A | Female | 22 |
| 893 | NE | NE | NE | NE | NE | 2 | NE | 5 | 2010 | D | A | Female | 35 |
| 892 | NE | NE | 4 | NE | NE | NE | 8 | NE | 2010 | E | A | Female | 18 |
| 891 | 4 | 3 | 5 | 2 | 2 | 3 | 5 | 5 | 2010 | E | A | Female | 20 |
| 890 | NE | NE | NE | NE | NE | NE | NE | NE | 2010 | E | A | Female | 17 |
| 889 | 7 | 7 | 8 | 5 | 7 | 7 | 8 | 5 | 2010 | E | A | Female | 20 |
| 888 | 6 | 7 | 9 | 6 | 6 | 6 | 8 | 9 | 2010 | E | A | Female | 26 |
| 887 | 6 | 7 | 7 | 6 | 6 | 7 | 7 | 7 | 2010 | E | A | Male | 23 |
| 886 | NE | NE | NE | 6 | 6 | NE | NE | NE | 2010 | E | A | Female | 26 |
| 885 | 5 | 5 | 8 | 6 | 6 | 8 | 5 | 5 | 2010 | E | A | Female | 47 |
| 884 | 7 | 8 | 10 | 6 | 8 | 7 | 10 | 9 | 2010 | E | A | Female | 22 |
| 883 | 7 | 9 | 8 | 10 | 7 | 8 | 8 | 9 | 2010 | E | A | Female | 21 |
| 882 | 4 | 6 | 7 | 3 | 7 | NE | 8 | 6 | 2010 | E | A | Male | 18 |
| 881 | 7 | 7 | 9 | 8 | 7 | 7 | 8 | 8 | 2010 | E | A | Female | 26 |
| 880 | W | W | W | W | W | W | W | W | 2010 | E | A | Female | 22 |
| 879 | 6 | 6 | 8 | 7 | 8 | 6 | 7 | 6 | 2010 | E | A | Female | 19 |
| 878 | 7 | 5 | 7 | 5 | 4 | 3 | 7 | 6 | 2010 | E | A | Female | 23 |
| 877 | 7 | 8 | 9 | 9 | 8 | 9 | 10 | 9 | 2010 | E | A | Female | 23 |
| 876 | 5 | 5 | 5 | 5 | 5 | 5 | 5 | 5 | 2010 | E | A | Female | 24 |
| 875 | W | W | W | W | W | W | W | W | 2010 | E | A | Female | 24 |
| 874 | 6 | 8 | 8 | 7 | 8 | NE | 10 | 7 | 2010 | E | A | Female | 22 |
| 873 | 7 | 6 | 9 | 7 | 7 | 5 | 10 | 7 | 2010 | E | A | Female | 22 |
| 872 | 6 | 6 | 9 | 6 | 7 | 5 | 10 | 5 | 2010 | E | A | Female | 23 |
| 871 | 7 | 7 | 7 | 5 | 8 | 9 | 10 | 8 | 2010 | E | A | Female | 21 |
| 870 | 7 | 6 | 9 | 6 | 6 | 8 | 9 | 9 | 2010 | E | A | Female | 34 |
| 869 | 6 | 6 | 6 | 6 | 5 | 6 | 6 | 6 | 2010 | E | A | Male | 29 |
| 868 | 7 | 8 | 10 | 10 | 10 | 10 | 10 | 10 | 2010 | E | A | Female | 31 |
| 867 | 5 | 5 | 5 | 5 | 5 | NE | 5 | NE | 2010 | E | A | Male | 20 |
| 866 | 5 | 5 | 7 | 5 | 5 | 7 | 7 | 7 | 2010 | M | B | Female | 19 |
| 865 | NE | NE | NE | NE | NE | NE | NE | NE | 2010 | M | B | Male | 19 |
| 864 | 3 | 5 | 3 | 5 | 6 | 6 | 4 | 3 | 2010 | M | B | Male | 20 |
| 863 | 8 | 8 | 8 | 10 | 10 | 6 | 8 | 9 | 2010 | M | B | Male | 19 |
| 862 | 5 | 6 | 7 | 8 | 5 | 5 | 5 | 6 | 2010 | M | B | Male | 21 |
| 861 | 8 | 7 | 8 | 10 | 10 | 9 | 8 | 8 | 2010 | M | B | Female | 18 |
| 860 | 3 | 3 | 2 | 2 | 3 | 2 | 4 | 4 | 2010 | M | B | Female | 19 |
| 859 | 6 | 7 | 6 | 8 | 8 | 6 | 7 | 7 | 2010 | M | B | Female | 19 |
| 858 | 3 | 3 | 3 | NE | 7 | 6 | NE | 3 | 2010 | M | B | Female | 19 |
| 857 | 3 | 3 | 2 | 2 | 6 | 2 | 2 | 3 | 2010 | M | B | Male | 20 |
| 856 | 5 | 5 | 6 | 8 | 6 | 6 | 9 | 7 | 2010 | M | B | Female | 22 |
| 855 | 3 | 5 | 5 | 5 | 5 | 5 | 7 | 7 | 2010 | M | B | Female | 19 |
| 854 | 5 | 7 | 5 | 7 | 8 | 7 | 8 | 7 | 2010 | M | B | Female | 18 |
| 853 | 5 | 5 | 7 | 7 | 7 | 5 | 7 | 6 | 2010 | M | B | Female | 18 |
| 852 | 8 | 5 | 5 | 5 | 5 | 5 | 8 | 7 | 2010 | M | B | Female | 20 |
| 851 | NE | NE | NE | NE | NE | NE | NE | NE | 2010 | M | B | Female | 18 |
| 850 | 5 | 5 | 5 | 5 | 5 | 5 | 8 | 6 | 2010 | M | B | Female | 19 |
| 849 | 5 | 5 | 5 | 5 | 8 | 5 | 6 | 6 | 2010 | M | B | Female | 18 |
| 848 | 5 | 6 | 6 | 7 | 8 | 6 | 6 | 7 | 2010 | M | B | Male | 29 |
| 847 | 5 | 7 | 7 | 6 | 9 | 6 | 5 | 8 | 2010 | M | B | Male | 22 |
| 846 | 3 | 5 | 2 | 2 | 5 | 2 | 3 | 3 | 2010 | M | B | Male | 19 |
| 845 | NE | NE | NE | NE | NE | NE | NE | NE | 2010 | M | B | Female | 22 |
| 844 | 5 | 6 | 6 | 5 | 6 | 5 | 5 | 5 | 2010 | M | B | Female | 19 |
| 843 | 3 | 5 | 2 | NE | 4 | 2 | 4 | 4 | 2010 | M | B | Female | 21 |
| 842 | 8 | 7 | 7 | 8 | 9 | 8 | 7 | 8 | 2010 | M | B | Female | 19 |
| 841 | 3 | 3 | 2 | NE | 4 | 2 | 4 | 2 | 2010 | M | B | Female | 19 |
| 840 | NE | NE | NE | NE | NE | NE | NE | NE | 2010 | M | A | Female | 19 |
| 839 | 7 | 7 | 6 | 6 | 8 | 6 | 7 | 6 | 2010 | M | A | Female | 21 |
| 838 | 5 | 3 | 4 | 2 | 5 | 3 | 3 | 3 | 2010 | M | A | Female | 20 |
| 837 | 5 | 6 | 6 | 6 | 7 | 3 | 7 | 5 | 2010 | M | A | Male | 19 |
| 836 | 3 | 5 | 5 | 5 | 7 | 2 | 4 | 6 | 2010 | M | A | Male | 20 |
| 835 | 3 | 3 | 4 | NE | 5 | 3 | 4 | 3 | 2010 | M | A | Female | 19 |
| 834 | 3 | 3 | 3 | 2 | 7 | 3 | 3 | 4 | 2010 | M | A | Female | 21 |
| 833 | 3 | 3 | 3 | 2 | 3 | 2 | 6 | 3 | 2010 | M | A | Female | 19 |
| 832 | 6 | 5 | 7 | 2 | 7 | 4 | 6 | 7 | 2010 | M | A | Female | 22 |
| 831 | 7 | 7 | 8 | 9 | 9 | 9 | 5 | 5 | 2010 | M | A | Female | 34 |
| 830 | 5 | 6 | 6 | 5 | 5 | 3 | 5 | 5 | 2010 | M | A | Female | 21 |
| 829 | NE | NE | NE | NE | NE | NE | NE | NE | 2010 | M | A | Male | 19 |
| 828 | 5 | 3 | 4 | 5 | 5 | NE | 5 | 4 | 2010 | M | A | Male | 20 |
| 827 | 5 | 5 | 7 | 5 | 5 | 7 | 8 | 8 | 2010 | M | A | Female | 19 |
| 826 | NE | NE | NE | NE | NE | NE | NE | NE | 2010 | M | A | Female | 19 |
| 825 | 6 | 5 | 6 | 6 | 8 | 5 | 6 | 6 | 2010 | M | A | Female | 20 |
| 824 | 6 | 3 | 4 | 6 | 5 | 2 | 4 | 5 | 2010 | M | A | Female | 20 |
| 823 | 5 | 6 | 4 | 2 | 8 | 4 | 4 | 5 | 2010 | M | A | Female | 21 |
| 822 | 5 | 5 | 8 | 7 | 5 | 6 | 8 | 9 | 2010 | M | A | Female | 20 |
| 821 | 6 | 7 | 6 | 8 | 8 | 5 | 7 | 5 | 2010 | M | A | Female | 27 |
| 820 | 6 | 5 | 6 | 10 | 9 | 5 | 8 | 8 | 2010 | M | A | Male | 19 |
| 819 | 5 | 6 | 8 | 8 | 9 | 6 | 7 | 6 | 2010 | M | A | Female | 19 |
| 818 | 3 | 5 | 3 | 5 | 5 | NE | 5 | 7 | 2010 | M | A | Female | 20 |
| 817 | 5 | 3 | NE | 2 | 5 | NE | 2 | 5 | 2010 | M | A | Female | 18 |
| 816 | NE | NE | NE | NE | NE | NE | NE | NE | 2010 | M | A | Female | 19 |
| 815 | 5 | 5 | 8 | 5 | 5 | 5 | 5 | 7 | 2010 | M | A | Male | 19 |
| 814 | 5 | 5 | 5 | 6 | 8 | 3 | 5 | 6 | 2010 | M | A | Female | 19 |
| 813 | 5 | 5 | 5 | 2 | NE | 3 | 5 | 3 | 2010 | M | A | Female | 20 |
| 812 | 8 | 10 | 7 | W | W | W | 8 | W | 2009 | D | A | Female | 31 |
| 811 | 5 | 6 | 4 | 5 | 5 | 2 | 5 | 7 | 2009 | D | A | Female | 23 |
| 810 | 7 | 9 | 6 | 10 | 8 | 7 | 9 | 5 | 2009 | D | A | Female | 26 |
| 809 | NE | NE | NE | NE | NE | NE | NE | NE | 2009 | D | A | Female | 21 |
| 808 | 5 | 5 | 4 | 8 | 4 | NE | 5 | 5 | 2009 | D | A | Female | 46 |
| 807 | NE | NE | NE | NE | NE | NE | NE | NE | 2009 | D | A | Female | 24 |
| 806 | NE | NE | NE | NE | NE | NE | NE | NE | 2009 | D | A | Female | 40 |
| 805 | NE | NE | NE | NE | NE | NE | NE | NE | 2009 | D | A | Female | 26 |
| 804 | W | W | 5 | W | 5 | W | 5 | W | 2009 | D | A | Male | 36 |
| 803 | NE | NE | NE | NE | NE | NE | NE | NE | 2009 | D | A | Female | 33 |
| 802 | 5 | 5 | 5 | 8 | 6 | 5 | 5 | 5 | 2009 | D | A | Male | 25 |
| 801 | W | W | W | W | W | W | W | W | 2009 | D | A | Female | 32 |
| 800 | 5 | NE | 5 | 5 | 5 | 5 | NE | NE | 2009 | D | A | Male | 36 |
| 799 | 5 | 5 | 5 | 3 | 5 | 4 | 5 | 5 | 2009 | D | A | Female | 23 |
| 798 | 2 | NE | 4 | NE | 2 | NE | NE | NE | 2009 | D | A | Female | 19 |
| 797 | 8 | 10 | 9 | 10 | 10 | W | 10 | 9 | 2009 | D | A | Female | 30 |
| 796 | 5 | 5 | 5 | 5 | 5 | 5 | 5 | 5 | 2009 | D | A | Male | 40 |
| 795 | NE | NE | NE | NE | NE | NE | NE | NE | 2009 | D | A | Female | 26 |
| 794 | NE | NE | NE | NE | NE | NE | NE | NE | 2009 | D | A | Male | 22 |
| 793 | 7 | 9 | 6 | 8 | 7 | 8 | 9 | 5 | 2009 | D | A | Male | 26 |
| 792 | 5 | NE | 4 | NE | NE | NE | 5 | 5 | 2009 | D | A | Female | 20 |
| 791 | 5 | 7 | 5 | 5 | 5 | 5 | 5 | 5 | 2009 | D | A | Male | 25 |
| 790 | 8 | 10 | 8 | 10 | 10 | W | 10 | 8 | 2009 | D | A | Female | 30 |
| 789 | 2 | NE | NE | NE | NE | NE | 5 | 5 | 2009 | D | A | Female | 23 |
| 788 | 5 | 10 | 9 | 5 | 5 | 8 | 5 | 5 | 2009 | D | A | Female | 28 |
| 787 | 5 | 5 | NE | NE | NE | 5 | NE | 5 | 2009 | D | A | Female | 23 |
| 786 | NE | NE | NE | NE | NE | NE | NE | NE | 2009 | D | A | Male | 23 |
| 785 | 6 | 8 | NE | NE | NE | 6 | 9 | 6 | 2009 | D | A | Female | 30 |
| 784 | 4 | NE | NE | NE | NE | NE | NE | NE | 2009 | D | A | Male | 19 |
| 783 | NE | NE | NE | NE | NE | NE | NE | NE | 2009 | D | A | Female | 28 |
| 782 | 5 | NE | 5 | 5 | 5 | 5 | NE | NE | 2009 | D | A | Female | 30 |
| 781 | 5 | 9 | 5 | 7 | 5 | 5 | 5 | 5 | 2009 | D | A | Female | 29 |
| 780 | 5 | 5 | 5 | 6 | 3 | 2 | 5 | 5 | 2009 | D | A | Male | 25 |
| 779 | W | W | W | W | W | W | W | W | 2009 | D | A | Female | 24 |
| 778 | 5 | NE | 2 | NE | NE | NE | 5 | NE | 2009 | D | A | Female | 21 |
| 777 | NE | NE | NE | NE | NE | NE | NE | NE | 2009 | D | A | Male | 24 |
| 776 | NE | NE | NE | NE | NE | 2 | NE | 5 | 2009 | D | A | Female | 24 |
| 775 | 5 | 5 | 5 | 3 | 5 | 3 | 5 | 5 | 2009 | D | A | Female | 26 |
| 774 | 5 | 7 | 4 | NE | NE | 5 | 5 | 5 | 2009 | D | A | Female | 32 |
| 773 | NE | NE | NE | NE | NE | NE | NE | NE | 2009 | D | A | Male | 27 |
| 772 | NE | NE | NE | NE | NE | NE | NE | NE | 2009 | D | A | Female | 24 |
| 771 | 6 | 5 | 5 | 7 | 7 | 8 | 8 | 5 | 2009 | D | A | Female | 23 |
| 770 | 5 | 5 | NE | 5 | 5 | NE | 5 | 5 | 2009 | D | A | Female | 20 |
| 769 | 2 | NE | NE | NE | NE | NE | NE | 3 | 2009 | D | A | Female | 20 |
| 768 | NE | NE | NE | NE | NE | NE | NE | NE | 2009 | D | A | Female | 37 |
| 767 | NE | NE | NE | NE | NE | NE | NE | NE | 2009 | D | A | Male | 29 |
| 766 | 5 | 7 | 5 | 5 | 5 | 5 | 5 | W | 2009 | D | A | Female | 30 |
| 765 | 5 | W | 5 | 8 | 7 | W | 5 | 5 | 2009 | D | A | Female | 23 |
| 764 | 8 | 7 | 4 | NE | 5 | 5 | 3 | NE | 2009 | D | A | Female | 19 |
| 763 | NE | NE | NE | NE | NE | NE | NE | NE | 2009 | D | A | Female | 21 |
| 762 | 2 | NE | NE | NE | NE | NE | NE | NE | 2009 | D | A | Male | 21 |
| 761 | 3 | NE | NE | NE | NE | 2 | NE | NE | 2009 | D | A | Male | 23 |
| 760 | 4 | NE | NE | NE | NE | 2 | NE | 5 | 2009 | D | A | Female | 21 |
| 759 | 2 | 5 | 5 | 5 | 5 | 4 | NE | 5 | 2009 | D | A | Female | 38 |
| 758 | W | W | W | W | W | NE | W | W | 2009 | D | A | Male | 39 |
| 757 | 7 | 8 | 7 | 5 | NE | 5 | 10 | 7 | 2009 | D | A | Female | 22 |
| 756 | 8 | 9 | 7 | 8 | 8 | 9 | 8 | 7 | 2009 | D | A | Female | 24 |
| 755 | NE | 4 | NE | 2 | NE | NE | NE | NE | 2009 | D | A | Female | 26 |
| 754 | NE | NE | NE | NE | NE | NE | NE | NE | 2009 | D | A | Female | 28 |
| 753 | 5 | 6 | NE | NE | NE | NE | NE | NE | 2009 | D | A | Male | 34 |
| 752 | NE | NE | NE | NE | NE | NE | NE | NE | 2009 | D | A | Female | 23 |
| 751 | 8 | 5 | 4 | 2 | 2 | 2 | 6 | NE | 2009 | D | A | Female | 32 |
| 750 | 4 | 7 | 4 | NE | NE | 2 | 6 | 6 | 2009 | D | A | Female | 32 |
| 749 | 7 | 8 | 7 | 9 | 8 | NE | 8 | 5 | 2009 | D | A | Female | 26 |
| 748 | 6 | 8 | 4 | NE | NE | 6 | 2 | 9 | 2009 | D | A | Male | 23 |
| 747 | 7 | 9 | 8 | 7 | 8 | 6 | 9 | 5 | 2009 | D | A | Female | 28 |
| 746 | NE | NE | NE | NE | NE | NE | NE | NE | 2009 | D | A | Female | 30 |
| 745 | NE | NE | NE | NE | NE | NE | NE | NE | 2009 | D | A | Female | 51 |
| 744 | 5 | 6 | 5 | NE | 4 | 5 | 5 | 5 | 2009 | D | A | Female | 30 |
| 743 | 5 | 8 | 5 | 7 | 6 | 5 | 5 | 5 | 2009 | D | A | Female | 21 |
| 742 | 6 | NE | 3 | NE | W | W | 6 | 6 | 2009 | D | A | Female | 22 |
| 741 | 7 | 8 | 7 | 2 | 6 | 7 | 8 | 5 | 2009 | D | A | Female | 30 |
| 740 | 5 | 8 | 5 | 9 | 6 | 5 | 5 | 5 | 2009 | D | A | Female | 40 |
| 739 | 5 | 5 | 5 | 5 | 5 | 5 | 5 | 7 | 2009 | D | A | Male | 19 |
| 738 | 2 | NE | NE | NE | NE | 2 | NE | NE | 2009 | D | A | Female | 30 |
| 737 | NE | NE | NE | NE | NE | NE | NE | NE | 2009 | D | A | Female | 19 |
| 736 | NE | NE | NE | NE | NE | NE | NE | NE | 2009 | D | A | Male | 24 |
| 735 | W | 8 | 7 | W | W | 9 | 9 | 7 | 2009 | D | A | Female | 45 |
| 734 | NE | NE | NE | NE | NE | NE | NE | NE | 2009 | D | A | Female | 23 |
| 733 | 2 | NE | NE | NE | NE | NE | NE | NE | 2009 | D | A | Female | 25 |
| 732 | 4 | NE | 2 | NE | NE | 2 | 3 | NE | 2009 | E | A | Male | 18 |
| 731 | 7 | 7 | 9 | 9 | 8 | 7 | 8 | 5 | 2009 | E | A | Male | 26 |
| 730 | 2 | 3 | 2 | NE | NE | NE | 3 | NE | 2009 | E | A | Male | 18 |
| 729 | 5 | 5 | 6 | 5 | 5 | 5 | 7 | 5 | 2009 | E | A | Male | 19 |
| 728 | 7 | 5 | 6 | 5 | 5 | 7 | 7 | 7 | 2009 | E | A | Female | 24 |
| 727 | 6 | 6 | 7 | 7 | 7 | 6 | 8 | 6 | 2009 | E | A | Female | 25 |
| 726 | 5 | NE | 5 | NE | NE | NE | 5 | 5 | 2009 | E | A | Female | 20 |
| 725 | 6 | 6 | W | W | W | W | 8 | 7 | 2009 | E | A | Female | 46 |
| 724 | 2 | 3 | 5 | NE | 3 | 2 | 4 | 5 | 2009 | E | A | Male | 23 |
| 723 | 5 | 6 | 7 | 5 | 7 | 5 | 5 | 7 | 2009 | E | A | Female | 23 |
| 722 | 5 | 5 | 6 | 6 | 5 | 3 | 8 | 5 | 2009 | E | A | Female | 20 |
| 721 | 8 | 7 | 9 | 10 | 9 | 8 | 9 | 8 | 2009 | E | A | Female | 24 |
| 720 | 5 | 6 | 8 | 7 | 8 | 6 | 9 | 8 | 2009 | E | A | Female | 19 |
| 719 | NE | NE | 2 | NE | NE | NE | 3 | NE | 2009 | E | A | Female | 21 |
| 718 | 5 | 6 | 7 | 8 | 8 | 7 | 8 | 7 | 2009 | E | A | Female | 21 |
| 717 | 6 | 4 | 5 | 2 | 5 | 7 | 7 | 8 | 2009 | E | A | Female | 23 |
| 716 | 5 | 5 | 5 | 5 | 7 | 5 | 7 | 5 | 2009 | E | A | Male | 25 |
| 715 | NE | NE | NE | NE | NE | NE | NE | NE | 2009 | E | A | Female | 20 |
| 714 | 5 | 5 | 6 | 5 | 5 | 6 | 5 | 7 | 2009 | E | A | Female | 25 |
| 713 | 7 | 5 | 5 | 6 | 5 | 6 | 5 | 5 | 2009 | E | A | Female | 26 |
| 712 | 5 | 6 | 5 | 9 | 5 | 5 | 5 | 5 | 2009 | E | A | Female | 18 |
| 711 | 5 | 5 | 3 | 5 | 5 | 3 | 2 | 3 | 2009 | M | B | Female | 18 |
| 710 | 7 | 7 | 7 | 10 | 8 | 7 | 6 | 7 | 2009 | M | B | Male | 19 |
| 709 | 3 | 5 | 6 | 6 | 6 | 6 | 5 | 5 | 2009 | M | B | Male | 19 |
| 708 | NE | 3 | 2 | NE | 2 | 2 | 2 | 2 | 2009 | M | B | Male | 19 |
| 707 | 8 | 6 | 5 | 8 | 7 | 7 | 8 | 4 | 2009 | M | B | Male | 19 |
| 706 | 3 | 3 | 3 | NE | 2 | 2 | 3 | 3 | 2009 | M | B | Male | 18 |
| 705 | 3 | 5 | 6 | 2 | 6 | 6 | 2 | 3 | 2009 | M | B | Female | 21 |
| 704 | 5 | 3 | 3 | NE | 5 | 5 | 5 | 3 | 2009 | M | B | Male | 20 |
| 703 | 6 | 5 | 5 | 7 | 5 | 6 | 5 | 4 | 2009 | M | B | Female | 22 |
| 702 | 3 | 2 | 2 | NE | 2 | 2 | 2 | NE | 2009 | M | B | Male | 22 |
| 701 | 7 | 3 | 3 | NE | NE | 2 | 4 | 3 | 2009 | M | B | Female | 21 |
| 700 | 3 | 6 | 6 | 7 | 8 | 6 | 7 | 5 | 2009 | M | B | Female | 19 |
| 699 | 2 | 6 | 6 | 6 | 6 | 5 | 2 | 3 | 2009 | M | B | Female | 18 |
| 698 | 5 | 6 | 9 | 6 | 7 | 8 | NE | 4 | 2009 | M | B | Female | 27 |
| 697 | 8 | 7 | 8 | 8 | 8 | 9 | 8 | 8 | 2009 | M | B | Male | 19 |
| 696 | 6 | 5 | 6 | 7 | 7 | 6 | 8 | 4 | 2009 | M | B | Male | 20 |
| 695 | 4 | 5 | 3 | 5 | 6 | 5 | 2 | 4 | 2009 | M | B | Male | 20 |
| 694 | 4 | 5 | 6 | 5 | 5 | 5 | 2 | 3 | 2009 | M | B | Female | 19 |
| 693 | 6 | 6 | 5 | 7 | 5 | 7 | 2 | 4 | 2009 | M | B | Female | 18 |
| 692 | 5 | 5 | 6 | 9 | 5 | 6 | 5 | 4 | 2009 | M | B | Female | 21 |
| 691 | 5 | 5 | 5 | 6 | 5 | 6 | 2 | 5 | 2009 | M | B | Male | 23 |
| 690 | 5 | 8 | 5 | 8 | 5 | 5 | 5 | 5 | 2009 | M | B | Female | 18 |
| 689 | 5 | 5 | 6 | 5 | 5 | 5 | 5 | 5 | 2009 | M | B | Male | 21 |
| 688 | 5 | NE | NE | NE | 5 | NE | NE | 3 | 2009 | M | A | Female | 19 |
| 687 | 5 | 6 | 6 | 8 | 7 | 7 | 3 | 6 | 2009 | M | A | Female | 52 |
| 686 | 3 | 3 | 2 | NE | 4 | 3 | NE | 3 | 2009 | M | A | Male | 18 |
| 685 | NE | NE | NE | NE | NE | NE | NE | NE | 2009 | M | A | Female | 20 |
| 684 | 6 | 6 | 7 | 8 | 6 | 6 | 8 | 4 | 2009 | M | A | Female | 22 |
| 683 | 6 | 5 | 6 | 3 | 5 | 6 | 5 | 5 | 2009 | M | A | Female | 20 |
| 682 | 7 | 6 | 3 | 2 | 7 | 7 | 2 | 4 | 2009 | M | A | Male | 20 |
| 681 | 4 | 3 | NE | NE | 5 | NE | NE | 3 | 2009 | M | A | Female | 20 |
| 680 | 5 | 5 | 4 | 5 | 5 | 6 | 5 | 5 | 2009 | M | A | Female | 20 |
| 679 | 5 | 3 | 3 | NE | NE | 2 | 3 | NE | 2009 | M | A | Female | 19 |
| 678 | 3 | 3 | 3 | 2 | 2 | NE | NE | NE | 2009 | M | A | Male | 19 |
| 677 | 6 | 5 | NE | 6 | 7 | NE | NE | NE | 2009 | M | A | Female | 18 |
| 676 | NE | NE | NE | 2 | 4 | NE | NE | NE | 2009 | M | A | Female | 19 |
| 675 | 3 | 3 | NE | 2 | 4 | NE | NE | 3 | 2009 | M | A | Female | 19 |
| 674 | 7 | 7 | 6 | 7 | 7 | 5 | 7 | 6 | 2009 | M | A | Male | 18 |
| 673 | 6 | 5 | 3 | 3 | 6 | 3 | 4 | 4 | 2009 | M | A | Female | 19 |
| 672 | 6 | 6 | 5 | 8 | 7 | 2 | 5 | 6 | 2009 | M | A | Female | 19 |
| 671 | W | W | 4 | 5 | 5 | W | W | 5 | 2009 | M | A | Female | 50 |
| 670 | 5 | 5 | 3 | 5 | 7 | NE | 2 | 5 | 2009 | M | A | Male | 18 |
| 669 | 6 | 5 | 5 | 5 | 6 | 4 | 5 | 6 | 2009 | M | A | Female | 18 |
| 668 | 8 | 6 | 4 | 5 | 6 | 6 | 5 | 4 | 2009 | M | A | Male | 18 |
| 667 | NE | 3 | 5 | NE | 2 | 2 | 2 | NE | 2009 | M | A | Female | 20 |
| 666 | 6 | 4 | 3 | NE | NE | NE | 3 | 3 | 2009 | M | A | Female | 19 |
| 665 | 5 | 6 | 5 | 8 | 8 | NE | 6 | 4 | 2009 | M | A | Male | 19 |
| 664 | 9 | W | W | 5 | 5 | W | 8 | W | 2008 | D | A | Female | 22 |
| 663 | NE | NE | NE | NE | NE | NE | NE | NE | 2008 | D | A | Female | 25 |
| 662 | NE | NE | NE | NE | NE | NE | NE | NE | 2008 | D | A | Male | 30 |
| 661 | 9 | W | 5 | W | W | W | 8 | NE | 2008 | D | A | Female | 25 |
| 660 | 6 | 5 | 5 | NE | 5 | 5 | 7 | 5 | 2008 | D | A | Female | 45 |
| 659 | NE | 6 | 5 | NE | NE | 3 | 5 | 5 | 2008 | D | A | Female | 29 |
| 658 | 6 | 5 | 5 | 5 | 7 | 5 | 8 | 5 | 2008 | D | A | Female | 22 |
| 657 | 9 | 6 | 6 | W | W | W | 9 | 5 | 2008 | D | A | Male | 24 |
| 656 | 8 | 6 | 6 | 2 | 6 | NE | 9 | NE | 2008 | D | A | Female | 22 |
| 655 | NE | NE | NE | NE | NE | NE | NE | NE | 2008 | D | A | Male | 23 |
| 654 | NE | NE | NE | NE | NE | NE | NE | NE | 2008 | D | A | Female | 22 |
| 653 | 6 | 5 | 7 | 3 | 5 | W | 8 | 6 | 2008 | D | A | Male | 39 |
| 652 | 5 | 5 | NE | NE | NE | NE | NE | 5 | 2008 | D | A | Female | 24 |
| 651 | 7 | NE | W | 4 | W | W | 9 | 3 | 2008 | D | A | Male | 24 |
| 650 | 8 | 9 | 8 | 6 | 10 | 10 | 9 | 7 | 2008 | D | A | Female | 28 |
| 649 | NE | NE | NE | NE | NE | NE | NE | NE | 2008 | D | A | Female | 29 |
| 648 | NE | NE | NE | NE | NE | NE | NE | NE | 2008 | D | A | Male | 31 |
| 647 | 10 | W | W | 6 | 8 | W | 9 | 5 | 2008 | D | A | Female | 27 |
| 646 | 5 | 7 | 6 | 6 | 6 | 2 | 5 | 5 | 2008 | D | A | Female | 21 |
| 645 | 5 | 5 | 5 | NE | 5 | NE | 5 | 5 | 2008 | D | A | Female | 21 |
| 644 | NE | NE | NE | NE | NE | NE | NE | NE | 2008 | D | A | Female | 22 |
| 643 | 5 | 8 | 5 | 8 | 7 | 5 | 7 | 5 | 2008 | D | A | Female | 30 |
| 642 | 9 | W | 6 | W | W | W | 9 | W | 2008 | D | A | Female | 28 |
| 641 | 6 | 6 | 6 | NE | W | W | 8 | 5 | 2008 | D | A | Male | 24 |
| 640 | 10 | 8 | 7 | 8 | 8 | 7 | 9 | 5 | 2008 | D | A | Female | 24 |
| 639 | 9 | 9 | 7 | 7 | 5 | 7 | 8 | 5 | 2008 | D | A | Female | 25 |
| 638 | 9 | 3 | 3 | 3 | W | W | 8 | 3 | 2008 | D | A | Female | 20 |
| 637 | 8 | 6 | 5 | 2 | NE | 2 | 5 | 5 | 2008 | D | A | Female | 25 |
| 636 | 9 | W | NE | W | W | W | 8 | 5 | 2008 | D | A | Female | 31 |
| 635 | 9 | 8 | 6 | 6 | 6 | 5 | 9 | 6 | 2008 | D | A | Male | 44 |
| 634 | NE | NE | NE | NE | NE | NE | NE | NE | 2008 | D | A | Female | 20 |
| 633 | 7 | 7 | 5 | 8 | 7 | 5 | 5 | 5 | 2008 | D | A | Male | 30 |
| 632 | W | W | W | W | W | W | W | W | 2008 | D | A | Female | 23 |
| 631 | NE | NE | NE | NE | NE | NE | NE | NE | 2008 | D | A | Male | 21 |
| 630 | 5 | 5 | NE | NE | 5 | 2 | NE | 5 | 2008 | D | A | Female | 22 |
| 629 | 10 | 10 | 9 | 7 | 10 | 9 | 9 | 8 | 2008 | D | A | Female | 28 |
| 628 | 10 | W | W | W | W | W | 9 | W | 2008 | D | A | Female | 29 |
| 627 | 8 | NE | 6 | NE | NE | 2 | 6 | 5 | 2008 | D | A | Female | 22 |
| 626 | NE | NE | NE | NE | NE | NE | NE | NE | 2008 | D | A | Female | 29 |
| 625 | NE | 5 | 5 | NE | NE | 5 | NE | 5 | 2008 | D | A | Female | 20 |
| 624 | 5 | 5 | 5 | 8 | 6 | 5 | 5 | 5 | 2008 | D | A | Female | 32 |
| 623 | W | W | W | W | W | W | W | W | 2008 | D | A | Male | 20 |
| 622 | 4 | 6 | 7 | NE | NE | NE | 4 | 6 | 2008 | D | A | Female | 37 |
| 621 | NE | W | 6 | W | 5 | W | 4 | 5 | 2008 | D | A | Female | 30 |
| 620 | 5 | 5 | 5 | 6 | 6 | 5 | 5 | 5 | 2008 | D | A | Female | 39 |
| 619 | NE | NE | NE | NE | NE | NE | NE | NE | 2008 | D | A | Male | 23 |
| 618 | NE | NE | NE | NE | NE | NE | NE | NE | 2008 | D | A | Male | 25 |
| 617 | NE | NE | NE | NE | NE | NE | NE | NE | 2008 | D | A | Female | 31 |
| 616 | 5 | 5 | 5 | 6 | 5 | 5 | 5 | 5 | 2008 | D | A | Female | 24 |
| 615 | NE | NE | NE | NE | NE | NE | NE | 5 | 2008 | D | A | Female | 21 |
| 614 | W | W | W | W | W | W | W | W | 2008 | D | A | Male | 23 |
| 613 | NE | NE | NE | NE | NE | NE | NE | NE | 2008 | D | A | Male | 33 |
| 612 | 5 | NE | NE | 5 | 5 | 5 | 5 | NE | 2008 | D | A | Female | 42 |
| 611 | 7 | W | 7 | W | W | 6 | 6 | 5 | 2008 | D | A | Female | 29 |
| 610 | W | W | W | W | W | W | W | W | 2008 | D | A | Female | 20 |
| 609 | 10 | 5 | 8 | 5 | 5 | 5 | 8 | 7 | 2008 | D | A | Female | 39 |
| 608 | 7 | 10 | 7 | 8 | 10 | 10 | 10 | 10 | 2008 | D | A | Female | 26 |
| 607 | 7 | 8 | 9 | 8 | 6 | 5 | 7 | 7 | 2008 | D | A | Female | 41 |
| 606 | 8 | 8 | 10 | 10 | 9 | 8 | 10 | 8 | 2008 | E | A | Female | 36 |
| 605 | 7 | 7 | 10 | 8 | 10 | 7 | 9 | 9 | 2008 | E | A | Female | 40 |
| 604 | 8 | 5 | 6 | 7 | 7 | 5 | 8 | 7 | 2008 | E | A | Male | 20 |
| 603 | 4 | 4 | 5 | 2 | 7 | 3 | 8 | 5 | 2008 | E | A | Male | 23 |
| 602 | 9 | 9 | 10 | 10 | 9 | 10 | 10 | 10 | 2008 | E | A | Female | 23 |
| 601 | 5 | 2 | 7 | 2 | 2 | 3 | 9 | 5 | 2008 | E | A | Female | 19 |
| 600 | 8 | 5 | 9 | 7 | 7 | 6 | 7 | 7 | 2008 | E | A | Female | 23 |
| 599 | 5 | 5 | 5 | 6 | 7 | 5 | 5 | 5 | 2008 | E | A | Male | 22 |
| 598 | 6 | 5 | 7 | 5 | 4 | 3 | 9 | 6 | 2008 | E | A | Female | 19 |
| 597 | 7 | 9 | 10 | 10 | 10 | 8 | 10 | 9 | 2008 | E | A | Female | 21 |
| 596 | W | W | W | W | 2 | NE | 3 | 3 | 2008 | E | A | Male | 19 |
| 595 | 5 | 8 | 8 | 5 | 7 | 7 | 8 | 8 | 2008 | E | A | Female | 23 |
| 594 | 6 | 6 | 8 | 7 | 7 | 6 | 9 | 8 | 2008 | E | A | Male | 23 |
| 593 | NE | 5 | 8 | 2 | 5 | NE | 9 | 7 | 2008 | E | A | Female | 25 |
| 592 | 5 | 5 | 5 | NE | 3 | 5 | 5 | 5 | 2008 | E | A | Male | 24 |
| 591 | 5 | 2 | 5 | 3 | 4 | NE | 8 | 5 | 2008 | E | A | Female | 17 |
| 590 | 5 | 4 | 5 | NE | 3 | 5 | 6 | 5 | 2008 | E | A | Female | 20 |
| 589 | NE | NE | 3 | NE | 3 | NE | 3 | NE | 2008 | E | A | Female | 19 |
| 588 | 9 | 8 | 10 | 9 | 9 | 10 | 9 | 9 | 2008 | E | A | Female | 22 |
| 587 | 5 | 6 | 6 | 5 | 5 | 5 | 5 | 7 | 2008 | M | B | Female | 19 |
| 586 | 9 | 5 | 6 | 7 | 7 | 7 | 6 | 5 | 2008 | M | B | Female | 18 |
| 585 | 5 | 5 | 5 | 5 | 5 | 2 | 5 | 5 | 2008 | M | B | Female | 23 |
| 584 | 3 | 5 | 5 | NE | 5 | 2 | 3 | 2 | 2008 | M | B | Female | 21 |
| 583 | 4 | 3 | 2 | NE | 2 | 2 | 3 | 2 | 2008 | M | B | Male | 21 |
| 582 | 7 | 5 | 6 | 6 | 7 | 7 | 5 | 5 | 2008 | M | B | Female | 19 |
| 581 | 8 | 6 | 7 | 8 | 7 | 5 | 7 | 7 | 2008 | M | B | Female | 19 |
| 580 | 5 | 5 | 5 | 2 | 7 | 6 | 6 | 6 | 2008 | M | B | Male | 19 |
| 579 | 5 | 5 | 5 | 9 | 7 | 5 | 7 | 6 | 2008 | M | B | Female | 19 |
| 578 | 6 | 4 | 2 | 2 | 7 | 2 | 4 | 2 | 2008 | M | B | Female | 20 |
| 577 | 5 | 5 | 2 | 2 | 5 | 2 | 3 | 2 | 2008 | M | B | Male | 22 |
| 576 | 8 | 7 | 6 | 7 | 6 | 6 | 5 | 6 | 2008 | M | B | Female | 20 |
| 575 | 7 | 5 | 5 | 5 | 5 | 5 | 6 | 6 | 2008 | M | B | Female | 20 |
| 574 | 7 | 2 | 5 | 4 | 6 | 6 | 7 | 5 | 2008 | M | B | Female | 17 |
| 573 | 9 | 8 | 8 | 10 | 9 | 8 | 9 | 7 | 2008 | M | B | Female | 24 |
| 572 | 6 | 5 | 6 | NE | 5 | 5 | 7 | 3 | 2008 | M | B | Male | 18 |
| 571 | 5 | 5 | 3 | NE | 6 | 5 | 5 | 6 | 2008 | M | B | Male | 20 |
| 570 | 8 | 7 | 5 | 8 | 9 | 2 | 8 | 7 | 2008 | M | A | Male | 18 |
| 569 | 5 | 5 | 3 | 8 | 5 | NE | 4 | 7 | 2008 | M | A | Male | 18 |
| 568 | 6 | 6 | 5 | 9 | 8 | 7 | 5 | 8 | 2008 | M | A | Male | 18 |
| 567 | 5 | 5 | 7 | 6 | 5 | 5 | 5 | 5 | 2008 | M | A | Female | 18 |
| 566 | 5 | 5 | 4 | 4 | NE | 6 | 4 | 5 | 2008 | M | A | Female | 22 |
| 565 | 6 | 6 | 7 | 7 | 7 | NE | 3 | 7 | 2008 | M | A | Female | 18 |
| 564 | 4 | 3 | 3 | 5 | NE | NE | 3 | 7 | 2008 | M | A | Female | 20 |
| 563 | 3 | 4 | 4 | W | W | W | W | NE | 2008 | M | A | Female | 21 |
| 562 | 3 | 3 | 3 | 3 | NE | 3 | 5 | 6 | 2008 | M | A | Female | 19 |
| 561 | 5 | 5 | 4 | 6 | 5 | 3 | 5 | 7 | 2008 | M | A | Female | 19 |
| 560 | 5 | 5 | 6 | 9 | 8 | 5 | 7 | 5 | 2008 | M | A | Female | 19 |
| 559 | 5 | 5 | 3 | 5 | 5 | NE | 5 | 6 | 2008 | M | A | Female | 19 |
| 558 | 5 | 5 | 7 | 6 | 6 | 5 | 5 | 7 | 2008 | M | A | Female | 18 |
| 557 | 6 | 5 | 5 | 7 | NE | 5 | 4 | 7 | 2008 | M | A | Female | 19 |
| 556 | 5 | 7 | 7 | 9 | 8 | 6 | 8 | 8 | 2008 | M | A | Male | 18 |
| 555 | 5 | 6 | 6 | 6 | 7 | NE | 6 | 7 | 2008 | M | A | Female | 18 |
| 554 | 7 | 7 | 10 | 9 | 9 | 8 | 9 | 9 | 2008 | M | A | Male | 18 |
| 553 | 6 | 6 | 9 | 9 | 8 | 6 | 8 | 9 | 2008 | M | A | Male | 17 |
| 552 | 2 | 3 | 5 | W | W | W | 3 | 5 | 2008 | M | A | Female | 19 |
| 551 | 2 | 2 | 2 | 2 | NE | NE | 2 | 4 | 2008 | M | A | Female | 19 |
| 550 | W | W | W | W | W | W | W | W | 2008 | M | A | Female | 19 |
| 549 | NE | NE | NE | NE | NE | NE | NE | NE | 2007 | D | A | Male | 22 |
| 548 | 4 | NE | NE | NE | NE | NE | 4 | NE | 2007 | D | A | Female | 20 |
| 547 | 4 | 5 | 5 | 5 | 5 | 5 | 9 | 5 | 2007 | D | A | Male | 23 |
| 546 | 6 | 5 | 6 | 9 | 5 | 6 | 9 | 6 | 2007 | D | A | Male | 46 |
| 545 | NE | NE | NE | NE | NE | NE | 4 | NE | 2007 | D | A | Female | 24 |
| 544 | NE | NE | 5 | NE | NE | NE | 5 | 5 | 2007 | D | A | Female | 28 |
| 543 | 5 | 4 | 5 | NE | NE | NE | 5 | 5 | 2007 | D | A | Female | 23 |
| 542 | NE | 6 | 6 | NE | NE | 7 | 2 | 7 | 2007 | D | A | Female | 21 |
| 541 | NE | 5 | NE | 5 | NE | 5 | NE | NE | 2007 | D | A | Male | 34 |
| 540 | NE | NE | NE | NE | NE | NE | NE | NE | 2007 | D | A | Female | 49 |
| 539 | NE | NE | 5 | NE | NE | NE | 5 | NE | 2007 | D | A | Female | 27 |
| 538 | 9 | 8 | 8 | 10 | 7 | 7 | 10 | 8 | 2007 | D | A | Male | 33 |
| 537 | NE | NE | NE | NE | NE | NE | NE | NE | 2007 | D | A | Male | 38 |
| 536 | NE | NE | NE | NE | NE | NE | NE | NE | 2007 | D | A | Female | 20 |
| 535 | 10 | 7 | 7 | 7 | 6 | 9 | 10 | 8 | 2007 | D | A | Male | 38 |
| 534 | NE | NE | NE | NE | NE | NE | NE | NE | 2007 | D | A | Female | 26 |
| 533 | NE | NE | NE | NE | NE | NE | NE | NE | 2007 | D | A | Female | 57 |
| 532 | NE | NE | NE | NE | NE | NE | NE | NE | 2007 | D | A | Female | 27 |
| 531 | 5 | 3 | 3 | 3 | 4 | NE | 5 | 5 | 2007 | D | A | Female | 20 |
| 530 | 7 | 5 | 5 | 2 | 5 | NE | 5 | 5 | 2007 | D | A | Female | 20 |
| 529 | NE | NE | NE | NE | NE | NE | NE | NE | 2007 | D | A | Male | 24 |
| 528 | NE | NE | NE | NE | NE | NE | NE | NE | 2007 | D | A | Female | 21 |
| 527 | NE | NE | NE | NE | 5 | 5 | 5 | 5 | 2007 | D | A | Female | 20 |
| 526 | NE | 5 | NE | 5 | NE | 5 | NE | NE | 2007 | D | A | Female | 28 |
| 525 | 10 | NE | 6 | NE | NE | 5 | 2 | 7 | 2007 | D | A | Female | 29 |
| 524 | NE | NE | NE | NE | NE | NE | 2 | NE | 2007 | D | A | Female | 19 |
| 523 | NE | NE | NE | NE | NE | NE | NE | NE | 2007 | D | A | Male | 27 |
| 522 | 5 | 5 | 5 | 4 | 5 | 5 | 5 | 5 | 2007 | D | A | Female | 22 |
| 521 | 5 | NE | NE | NE | NE | NE | 5 | 5 | 2007 | D | A | Female | 24 |
| 520 | NE | NE | 5 | 2 | 3 | 6 | 5 | 5 | 2007 | D | A | Male | 29 |
| 519 | 8 | NE | 7 | NE | NE | 2 | 8 | 8 | 2007 | D | A | Female | 26 |
| 518 | NE | NE | NE | NE | NE | NE | NE | NE | 2007 | D | A | Female | 28 |
| 517 | NE | NE | NE | NE | NE | 2 | 4 | 7 | 2007 | D | A | Female | 24 |
| 516 | 9 | 7 | 7 | 8 | 7 | 9 | 10 | 8 | 2007 | D | A | Female | 35 |
| 515 | NE | NE | NE | NE | NE | NE | 4 | NE | 2007 | D | A | Female | 28 |
| 514 | NE | NE | NE | 5 | 5 | NE | 5 | 5 | 2007 | D | A | Female | 28 |
| 513 | 10 | 6 | 6 | NE | NE | 6 | 8 | 7 | 2007 | D | A | Female | 31 |
| 512 | 10 | 6 | 8 | 9 | 8 | 7 | 9 | 8 | 2007 | D | A | Female | 39 |
| 511 | NE | 5 | NE | 5 | 5 | NE | NE | NE | 2007 | D | A | Female | 44 |
| 510 | NE | NE | NE | NE | NE | NE | NE | NE | 2007 | D | A | Female | 22 |
| 509 | NE | NE | NE | NE | NE | NE | NE | NE | 2007 | D | A | Male | 33 |
| 508 | 8 | 8 | 8 | 9 | 9 | 9 | 9 | 8 | 2007 | D | A | Female | 35 |
| 507 | 5 | 5 | 7 | 6 | 6 | 8 | 7 | 8 | 2007 | D | A | Female | 19 |
| 506 | NE | NE | NE | NE | NE | 2 | 4 | 5 | 2007 | D | A | Female | 29 |
| 505 | 9 | 5 | 7 | NE | NE | 5 | 7 | 7 | 2007 | D | A | Female | 38 |
| 504 | NE | NE | NE | NE | NE | NE | NE | NE | 2007 | D | A | Male | 22 |
| 503 | 7 | 5 | 5 | 5 | 5 | 5 | 8 | 5 | 2007 | D | A | Female | 33 |
| 502 | NE | NE | NE | NE | NE | NE | NE | 5 | 2007 | D | A | Female | 26 |
| 501 | 9 | 5 | 5 | NE | NE | 6 | 8 | 5 | 2007 | D | A | Female | 23 |
| 500 | NE | NE | NE | NE | NE | 2 | NE | NE | 2007 | D | A | Male | 19 |
| 499 | 5 | 5 | 5 | 7 | NE | 5 | 5 | 5 | 2007 | D | A | Female | 23 |
| 498 | 5 | 5 | 4 | 5 | 5 | 5 | 5 | NE | 2007 | D | A | Female | 41 |
| 497 | 9 | 9 | 10 | 9 | 9 | 10 | 10 | 5 | 2007 | D | A | Female | 28 |
| 496 | NE | NE | 5 | NE | NE | NE | 5 | NE | 2007 | D | A | Female | 20 |
| 495 | 5 | 5 | 5 | 5 | 5 | 5 | 6 | 5 | 2007 | E | A | Female | 20 |
| 494 | 5 | 5 | 5 | 5 | 5 | 2 | 7 | 5 | 2007 | E | A | Female | 20 |
| 493 | 5 | 5 | 7 | 6 | 7 | 5 | 8 | 6 | 2007 | E | A | Female | 19 |
| 492 | 6 | 7 | 6 | 7 | 9 | 2 | 7 | 6 | 2007 | E | A | Female | 21 |
| 491 | 5 | 5 | 5 | 7 | 5 | 6 | 8 | 5 | 2007 | E | A | Female | 20 |
| 490 | 5 | 5 | 6 | 7 | 5 | 5 | 5 | 5 | 2007 | E | A | Female | 20 |
| 489 | 5 | 5 | 7 | 5 | 5 | 5 | 7 | 5 | 2007 | E | A | Female | 19 |
| 488 | 5 | 5 | 5 | 5 | 6 | 5 | 8 | 6 | 2007 | E | A | Female | 20 |
| 487 | 4 | 5 | 6 | 3 | 3 | 5 | 5 | 5 | 2007 | E | A | Female | 21 |
| 486 | 6 | 5 | 6 | 4 | 7 | 5 | 5 | 5 | 2007 | E | A | Male | 23 |
| 485 | 5 | 5 | 5 | 5 | 5 | 5 | 7 | 6 | 2007 | E | A | Male | 20 |
| 484 | 5 | 6 | 6 | 8 | 8 | 5 | 5 | 7 | 2007 | E | A | Male | 23 |
| 483 | 5 | 5 | 5 | 2 | 3 | 5 | 5 | 5 | 2007 | E | A | Female | 23 |
| 482 | 4 | 5 | 5 | NE | 3 | 5 | 5 | 6 | 2007 | E | A | Male | 19 |
| 481 | 3 | 3 | 2 | NE | NE | 6 | 4 | NE | 2007 | M | B | Female | 18 |
| 480 | 2 | 6 | 5 | 6 | 6 | 2 | 4 | NE | 2007 | M | B | Female | 19 |
| 479 | 4 | 5 | 5 | 4 | 2 | 5 | 4 | NE | 2007 | M | B | Female | 20 |
| 478 | 4 | 6 | 9 | 8 | 9 | 9 | 6 | 7 | 2007 | M | B | Female | 19 |
| 477 | 7 | 5 | 2 | W | W | W | 3 | 5 | 2007 | M | B | Female | 23 |
| 476 | 5 | 5 | 7 | 5 | 7 | 7 | 6 | 5 | 2007 | M | B | Male | 19 |
| 475 | 7 | 5 | 5 | 3 | 2 | 6 | 4 | 3 | 2007 | M | B | Female | 18 |
| 474 | NE | 3 | 2 | 3 | 3 | 2 | NE | NE | 2007 | M | B | Female | 18 |
| 473 | 3 | 3 | 2 | NE | 2 | 2 | 3 | 2 | 2007 | M | B | Female | 19 |
| 472 | 4 | 3 | 2 | NE | 2 | 2 | 4 | 2 | 2007 | M | B | Female | 18 |
| 471 | 4 | 6 | 6 | 5 | 6 | 2 | 4 | NE | 2007 | M | B | Female | 19 |
| 470 | 3 | 3 | NE | 3 | 3 | 2 | 3 | 3 | 2007 | M | B | Female | 20 |
| 469 | NE | 4 | 3 | 3 | 4 | 2 | 2 | NE | 2007 | M | B | Female | 17 |
| 468 | 6 | 6 | 7 | 6 | 7 | 8 | 7 | 7 | 2007 | M | B | Male | 19 |
| 467 | 4 | 6 | 8 | 5 | 4 | 7 | 4 | 6 | 2007 | M | B | Female | 17 |
| 466 | 2 | 5 | 2 | 4 | 4 | 2 | 3 | 3 | 2007 | M | B | Female | 18 |
| 465 | 5 | 6 | 5 | 6 | 5 | 2 | 6 | 5 | 2007 | M | A | Male | 19 |
| 464 | 5 | 6 | 5 | 7 | 5 | 5 | 6 | 5 | 2007 | M | A | Female | 20 |
| 463 | 5 | 5 | NE | NE | NE | NE | NE | 5 | 2007 | M | A | Female | 20 |
| 462 | 5 | 4 | NE | 5 | 5 | NE | 5 | 5 | 2007 | M | A | Female | 22 |
| 461 | 6 | 6 | 4 | 2 | 2 | 3 | 4 | 5 | 2007 | M | A | Female | 17 |
| 460 | 7 | 6 | 5 | 7 | W | 6 | 6 | 8 | 2007 | M | A | Female | 20 |
| 459 | 5 | 5 | 9 | 5 | 5 | 8 | 5 | 5 | 2007 | M | A | Female | 20 |
| 458 | 6 | 6 | 7 | 5 | 7 | 7 | 6 | 7 | 2007 | M | A | Female | 24 |
| 457 | 7 | 6 | 6 | 10 | 9 | 2 | 7 | 7 | 2007 | M | A | Male | 18 |
| 456 | 5 | 6 | 7 | 9 | 8 | 7 | 6 | 6 | 2007 | M | A | Male | 18 |
| 455 | 6 | 7 | NE | W | 4 | W | W | 7 | 2007 | M | A | Male | 18 |
| 454 | 5 | 5 | 5 | 5 | 7 | 3 | 5 | 5 | 2007 | M | A | Female | 20 |
| 453 | 6 | 6 | 6 | 7 | 6 | 7 | 5 | 8 | 2007 | M | A | Male | 23 |
| 452 | 8 | 10 | 8 | 10 | 9 | 9 | 8 | 10 | 2007 | M | A | Female | 20 |
| 451 | 7 | 5 | 7 | 5 | 8 | 7 | 5 | 8 | 2007 | M | A | Female | 23 |
| 450 | 6 | 5 | 5 | 6 | 6 | 6 | 2 | 7 | 2007 | M | A | Female | 40 |
| 449 | NE | NE | NE | NE | NE | NE | NE | NE | 2006 | D | A | Female | 20 |
| 448 | NE | NE | NE | NE | NE | NE | NE | NE | 2006 | D | A | Female | 23 |
| 447 | 8 | NE | 6 | NE | NE | NE | 7 | 6 | 2006 | D | A | Female | 22 |
| 446 | NE | NE | NE | NE | NE | NE | NE | NE | 2006 | D | A | Male | 38 |
| 445 | NE | NE | NE | NE | NE | NE | NE | NE | 2006 | D | A | Male | 23 |
| 444 | NE | NE | 7 | NE | NE | NE | 9 | NE | 2006 | D | A | Female | 26 |
| 443 | NE | NE | NE | NE | NE | NE | NE | NE | 2006 | D | A | Female | 20 |
| 442 | 2 | NE | NE | NE | NE | 2 | 8 | 5 | 2006 | D | A | Female | 28 |
| 441 | NE | NE | NE | NE | NE | NE | 3 | NE | 2006 | D | A | Female | 25 |
| 440 | 4 | 5 | 5 | 5 | 5 | 5 | 8 | 5 | 2006 | D | A | Female | 44 |
| 439 | NE | NE | NE | NE | NE | NE | 2 | NE | 2006 | D | A | Male | 25 |
| 438 | 2 | NE | NE | NE | NE | 2 | 3 | NE | 2006 | D | A | Male | 21 |
| 437 | NE | NE | NE | NE | NE | NE | NE | NE | 2006 | D | A | Male | 20 |
| 436 | 5 | 5 | 5 | 5 | 5 | NE | 5 | NE | 2006 | D | A | Female | 21 |
| 435 | NE | NE | NE | NE | NE | NE | NE | NE | 2006 | D | A | Female | 29 |
| 434 | NE | 5 | NE | NE | 5 | NE | 5 | 5 | 2006 | D | A | Female | 33 |
| 433 | NE | NE | NE | NE | NE | NE | NE | NE | 2006 | D | A | Male | 37 |
| 432 | 7 | NE | NE | NE | NE | NE | 9 | 5 | 2006 | D | A | Female | 19 |
| 431 | 5 | 9 | 5 | 7 | 8 | 9 | 9 | 7 | 2006 | D | A | Female | 26 |
| 430 | 2 | NE | 2 | NE | NE | 2 | 2 | NE | 2006 | D | A | Female | 20 |
| 429 | 7 | 5 | 5 | NE | 6 | 5 | 9 | 5 | 2006 | D | A | Female | 19 |
| 428 | 5 | 5 | 5 | 2 | 5 | 2 | 7 | 6 | 2006 | D | A | Female | 21 |
| 427 | 7 | 3 | 2 | 4 | 4 | 2 | 9 | 6 | 2006 | D | A | Female | 23 |
| 426 | 2 | 4 | 6 | NE | NE | 2 | 8 | 8 | 2006 | D | A | Male | 28 |
| 425 | NE | NE | NE | NE | NE | NE | NE | NE | 2006 | D | A | Female | 19 |
| 424 | NE | NE | NE | NE | NE | NE | NE | NE | 2006 | D | A | Female | 32 |
| 423 | NE | NE | NE | NE | NE | NE | NE | NE | 2006 | D | A | Female | 27 |
| 422 | 5 | 5 | 5 | 5 | 5 | 2 | 4 | 5 | 2006 | D | A | Female | 19 |
| 421 | 4 | 3 | 3 | NE | NE | NE | 4 | NE | 2006 | D | A | Male | 22 |
| 420 | 5 | 5 | 5 | NE | 5 | NE | 5 | NE | 2006 | D | A | Female | 28 |
| 419 | NE | NE | NE | NE | NE | NE | NE | NE | 2006 | D | A | Female | 35 |
| 418 | NE | 8 | 8 | 6 | 8 | 7 | NE | 9 | 2006 | D | A | Female | 32 |
| 417 | NE | NE | NE | NE | NE | NE | NE | NE | 2006 | D | A | Female | 20 |
| 416 | NE | NE | NE | NE | NE | NE | NE | 5 | 2006 | D | A | Female | 25 |
| 415 | 5 | 5 | 5 | NE | 2 | 5 | 5 | 5 | 2006 | D | A | Female | 22 |
| 414 | NE | NE | NE | NE | NE | NE | NE | NE | 2006 | D | A | Female | 25 |
| 413 | NE | NE | NE | NE | NE | NE | NE | NE | 2006 | D | A | Female | 34 |
| 412 | 8 | 5 | NE | 5 | 5 | NE | 9 | 5 | 2006 | D | A | Female | 40 |
| 411 | 8 | 8 | 9 | 8 | 9 | 9 | 9 | 9 | 2006 | D | A | Female | 27 |
| 410 | NE | NE | NE | NE | NE | NE | NE | NE | 2006 | D | A | Female | 19 |
| 409 | 5 | 5 | 5 | 5 | NE | NE | NE | 5 | 2006 | D | A | Male | 20 |
| 408 | NE | NE | NE | NE | NE | NE | NE | NE | 2006 | D | A | Male | 24 |
| 407 | NE | NE | NE | NE | NE | NE | NE | NE | 2006 | D | A | Male | 28 |
| 406 | 6 | 6 | 7 | 6 | 7 | 5 | 7 | 6 | 2006 | E | A | Female | 25 |
| 405 | 6 | 7 | 8 | 7 | 10 | 6 | 10 | 6 | 2006 | E | A | Female | 19 |
| 404 | 4 | 7 | 8 | 4 | 4 | 7 | 4 | NE | 2006 | E | A | Male | 24 |
| 403 | 4 | NE | 5 | 2 | 5 | 3 | 8 | 3 | 2006 | E | A | Female | 19 |
| 402 | 4 | 5 | 6 | 5 | 6 | 7 | 7 | 5 | 2006 | E | A | Female | 20 |
| 401 | 7 | 8 | 9 | 8 | 8 | 8 | 8 | 7 | 2006 | E | A | Female | 33 |
| 400 | 4 | 7 | 7 | 7 | 9 | 7 | 7 | 5 | 2006 | E | A | Female | 20 |
| 399 | 4 | 7 | 6 | 7 | 9 | 6 | 6 | 5 | 2006 | E | A | Female | 25 |
| 398 | 4 | 3 | 5 | 4 | 3 | 4 | 5 | 3 | 2006 | E | A | Female | 19 |
| 397 | 6 | 8 | 10 | 9 | 10 | 8 | 10 | 5 | 2006 | E | A | Male | 21 |
| 396 | 4 | 5 | 7 | 3 | 5 | 3 | 3 | 3 | 2006 | M | B | Female | 19 |
| 395 | 3 | 6 | 5 | 4 | 8 | 5 | 4 | 5 | 2006 | M | B | Male | 22 |
| 394 | 5 | 5 | 5 | 2 | 5 | 3 | 3 | NE | 2006 | M | B | Female | 19 |
| 393 | 8 | 6 | 2 | 5 | 6 | 2 | 2 | 3 | 2006 | M | B | Female | 19 |
| 392 | 5 | 6 | 6 | 4 | 4 | 6 | 5 | 5 | 2006 | M | B | Male | 19 |
| 391 | 3 | 5 | 3 | 2 | NE | 5 | NE | 2 | 2006 | M | B | Female | 19 |
| 390 | 5 | 4 | 3 | 4 | 4 | 3 | 4 | 3 | 2006 | M | B | Female | 18 |
| 389 | 6 | 5 | 2 | 2 | 4 | 2 | 4 | 3 | 2006 | M | B | Female | 18 |
| 388 | 5 | 5 | 5 | 5 | 6 | 5 | 6 | 4 | 2006 | M | B | Female | 18 |
| 387 | 2 | 3 | 2 | NE | NE | 2 | NE | NE | 2006 | M | B | Female | 19 |
| 386 | 7 | 5 | 2 | NE | NE | 5 | 2 | 2 | 2006 | M | B | Female | 19 |
| 385 | 6 | 7 | 2 | 2 | 6 | 2 | 5 | 2 | 2006 | M | B | Male | 20 |
| 384 | 5 | 5 | 5 | 4 | 4 | 5 | 5 | 5 | 2006 | M | B | Male | 17 |
| 383 | 5 | 5 | 5 | 5 | 5 | 6 | 5 | 5 | 2006 | M | B | Male | 20 |
| 382 | 5 | 6 | 6 | 6 | 7 | 6 | 7 | 5 | 2006 | M | B | Female | 19 |
| 381 | 3 | 4 | 2 | NE | NE | 2 | NE | NE | 2006 | M | B | Female | 19 |
| 380 | 6 | 6 | 5 | NE | NE | 3 | 2 | 2 | 2006 | M | B | Male | 19 |
| 379 | 5 | 5 | 5 | 7 | 7 | 7 | 2 | 5 | 2006 | M | B | Female | 22 |
| 378 | 7 | 5 | 5 | 5 | 7 | 5 | 5 | 7 | 2006 | M | A | Female | 19 |
| 377 | 7 | 4 | 3 | NE | 4 | 3 | 3 | 4 | 2006 | M | A | Female | 19 |
| 376 | 8 | 8 | 8 | 10 | 9 | 8 | 7 | 9 | 2006 | M | A | Male | 23 |
| 375 | 3 | 3 | 2 | NE | NE | NE | 2 | NE | 2006 | M | A | Female | 19 |
| 374 | 3 | 3 | 2 | NE | NE | NE | 2 | NE | 2006 | M | A | Female | 19 |
| 373 | 8 | 6 | 3 | 2 | 4 | 2 | 3 | 4 | 2006 | M | A | Female | 20 |
| 372 | 5 | 5 | 5 | 9 | 5 | 5 | 5 | 9 | 2006 | M | A | Female | 21 |
| 371 | 7 | 6 | 6 | 9 | 6 | 3 | 6 | 6 | 2006 | M | A | Female | 18 |
| 370 | 6 | 4 | 3 | NE | 3 | NE | 2 | 5 | 2006 | M | A | Female | 20 |
| 369 | 9 | 8 | 7 | 9 | 8 | 6 | 5 | 8 | 2006 | M | A | Female | 19 |
| 368 | 8 | 6 | 3 | 6 | 7 | 4 | 6 | 6 | 2006 | M | A | Female | 19 |
| 367 | 7 | 6 | 5 | NE | 7 | 2 | 4 | 6 | 2006 | M | A | Female | 19 |
| 366 | 7 | 6 | 4 | NE | 6 | 5 | 5 | 6 | 2006 | M | A | Female | 19 |
| 365 | 6 | 6 | 3 | 5 | 6 | 2 | 3 | 6 | 2006 | M | A | Female | 18 |
| 364 | 7 | 7 | 6 | 8 | 8 | 7 | 6 | 8 | 2006 | M | A | Female | 25 |
| 363 | 7 | 6 | 3 | 5 | 6 | 6 | 8 | 7 | 2006 | M | A | Female | 20 |
| 362 | 7 | 5 | 5 | 6 | 3 | 3 | 5 | 6 | 2006 | M | A | Male | 19 |
| 361 | 7 | 5 | 3 | NE | 7 | 3 | 2 | 6 | 2006 | M | A | Female | 20 |
| 360 | 7 | 6 | 6 | 8 | 9 | 6 | 7 | 7 | 2006 | M | A | Female | 20 |
| 359 | 8 | 6 | 5 | 6 | 5 | 3 | 5 | 6 | 2006 | M | A | Female | 19 |
| 358 | 4 | 3 | 2 | NE | NE | NE | 2 | 3 | 2006 | M | A | Female | 19 |
| 357 | 5 | 4 | 2 | NE | NE | NE | 2 | 4 | 2006 | M | A | Female | 18 |
| 356 | 8 | 6 | 5 | 6 | 6 | 4 | 3 | 7 | 2006 | M | A | Female | 19 |
| 355 | 6 | 5 | 5 | 6 | 6 | 6 | 2 | 7 | 2006 | M | A | Female | 39 |
| 354 | NE | NE | NE | NE | NE | NE | NE | NE | 2005 | D | A | Female | - |
| 353 | NE | NE | 2 | NE | NE | NE | NE | NE | 2005 | D | A | Female | - |
| 352 | NE | 2 | 3 | NE | NE | 3 | 3 | NE | 2005 | D | A | Female | - |
| 351 | NE | NE | 2 | NE | NE | NE | 3 | NE | 2005 | D | A | Female | - |
| 350 | NE | NE | NE | NE | NE | NE | NE | NE | 2005 | D | A | Female | - |
| 349 | 6 | NE | 6 | NE | 6 | NE | 6 | NE | 2005 | D | A | Female | - |
| 348 | NE | NE | NE | NE | NE | NE | NE | NE | 2005 | D | A | Female | - |
| 347 | NE | NE | 3 | NE | NE | NE | 2 | NE | 2005 | D | A | Female | - |
| 346 | NE | 2 | 3 | NE | NE | 4 | 2 | NE | 2005 | D | A | Male | - |
| 345 | 3 | 3 | 2 | NE | NE | NE | 2 | NE | 2005 | D | A | Female | - |
| 344 | NE | NE | NE | NE | NE | NE | NE | NE | 2005 | D | A | Female | - |
| 343 | NE | NE | NE | NE | NE | NE | NE | NE | 2005 | D | A | Female | - |
| 342 | NE | NE | NE | NE | NE | NE | NE | NE | 2005 | D | A | Female | - |
| 341 | 6 | 6 | 6 | 6 | W | 6 | 6 | 6 | 2005 | D | A | Male | - |
| 340 | NE | NE | NE | NE | NE | NE | NE | NE | 2005 | D | A | Female | - |
| 339 | NE | NE | NE | NE | NE | NE | NE | NE | 2005 | D | A | Male | - |
| 338 | NE | NE | NE | NE | NE | NE | NE | NE | 2005 | D | A | Male | - |
| 337 | 9 | NE | 8 | NE | NE | NE | NE | NE | 2005 | D | A | Female | - |
| 336 | NE | NE | NE | NE | NE | NE | NE | NE | 2005 | D | A | Female | - |
| 335 | 8 | 8 | 8 | 9 | 9 | 10 | 9 | 8 | 2005 | D | A | Male | - |
| 334 | 9 | 7 | 8 | 9 | 7 | 9 | 10 | 7 | 2005 | D | A | Female | - |
| 333 | NE | NE | NE | NE | NE | NE | NE | NE | 2005 | D | A | Female | - |
| 332 | NE | NE | NE | NE | NE | NE | NE | NE | 2005 | D | A | Female | - |
| 331 | NE | 6 | 6 | NE | 6 | NE | NE | NE | 2005 | D | A | Female | - |
| 330 | NE | NE | 3 | NE | NE | NE | 4 | NE | 2005 | D | A | Female | - |
| 329 | NE | 2 | 2 | NE | NE | 4 | 4 | NE | 2005 | D | A | Female | - |
| 328 | 10 | 9 | 8 | 9 | 8 | 10 | 8 | 8 | 2005 | D | A | Male | - |
| 327 | 10 | 6 | 6 | 8 | 5 | 7 | 8 | 5 | 2005 | D | A | Female | - |
| 326 | 6 | 6 | 6 | NE | 6 | NE | 6 | NE | 2005 | D | A | Female | - |
| 325 | NE | NE | NE | NE | NE | NE | 3 | NE | 2005 | D | A | Female | - |
| 324 | 9 | 6 | 8 | 3 | 5 | 8 | 6 | 7 | 2005 | D | A | Female | - |
| 323 | NE | NE | NE | NE | NE | NE | NE | NE | 2005 | D | A | Female | - |
| 322 | 6 | 6 | 6 | 6 | 6 | 6 | 6 | 6 | 2005 | D | A | Female | - |
| 321 | 2 | NE | NE | NE | NE | 3 | 2 | NE | 2005 | D | A | Female | - |
| 320 | NE | NE | NE | NE | NE | NE | NE | NE | 2005 | D | A | Male | - |
| 319 | 6 | 6 | 7 | 4 | 6 | 7 | 8 | 5 | 2005 | D | A | Female | - |
| 318 | 6 | 6 | 6 | NE | NE | 6 | 6 | NE | 2005 | D | A | Female | - |
| 317 | 8 | 6 | 7 | 8 | 8 | 10 | 9 | 8 | 2005 | D | A | Female | - |
| 316 | 8 | 8 | 9 | 9 | 9 | 9 | 9 | 7 | 2005 | D | A | Female | - |
| 315 | NE | NE | NE | NE | NE | NE | NE | NE | 2005 | D | A | Male | - |
| 314 | 4 | 6 | 6 | 6 | 4 | 6 | 6 | 7 | 2005 | E | A | Male | - |
| 313 | 6 | 4 | 3 | 3 | 6 | 4 | 6 | 5 | 2005 | E | A | Male | - |
| 312 | 2 | NE | NE | 2 | NE | 2 | NE | 3 | 2005 | E | A | Female | - |
| 311 | 6 | 5 | 6 | 6 | 5 | 6 | 6 | 6 | 2005 | E | A | Female | - |
| 310 | 4 | 4 | 5 | 6 | 5 | 5 | 6 | 6 | 2005 | E | A | Female | - |
| 309 | NE | 6 | 6 | NE | 6 | 6 | 6 | 6 | 2005 | E | A | Female | - |
| 308 | NE | NE | NE | NE | NE | NE | NE | NE | 2005 | E | A | Female | - |
| 307 | 6 | 4 | 5 | 7 | 6 | 6 | 5 | 7 | 2005 | E | A | Female | - |
| 306 | 6 | 6 | 7 | 6 | 7 | 6 | 6 | 6 | 2005 | E | A | Male | - |
| 305 | 7 | 9 | 7 | 6 | 6 | 8 | 6 | 7 | 2005 | E | A | Female | - |
| 304 | 5 | 4 | 6 | 5 | 4 | 5 | 6 | 7 | 2005 | E | A | Female | - |
| 303 | 3 | NE | 3 | NE | NE | NE | 4 | NE | 2005 | E | A | Male | - |
| 302 | 6 | 4 | 6 | 3 | 8 | 7 | 7 | 7 | 2005 | E | A | Female | - |
| 301 | 6 | 6 | 5 | 5 | 6 | 6 | 6 | 6 | 2005 | E | A | Female | - |
| 300 | 7 | 8 | 9 | 7 | 8 | 7 | 8 | 8 | 2005 | E | A | Female | - |
| 299 | 7 | 6 | 6 | 6 | 5 | 4 | 6 | 6 | 2005 | E | A | Female | - |
| 298 | 7 | 6 | 6 | 6 | 7 | 6 | 6 | 6 | 2005 | E | A | Female | - |
| 297 | 8 | 6 | 7 | 6 | 6 | 6 | 6 | 6 | 2005 | E | A | Male | - |
| 296 | NE | NE | NE | NE | NE | NE | 3 | NE | 2005 | E | A | Male | - |
| 295 | 5 | 6 | 8 | 9 | 8 | 8 | 8 | 5 | 2005 | M | B | Female | - |
| 294 | 6 | 6 | 9 | 7 | 7 | 6 | 5 | 7 | 2005 | M | B | Female | - |
| 293 | 5 | 7 | 7 | NE | 5 | 2 | 3 | 2 | 2005 | M | B | Female | - |
| 292 | 6 | 7 | 6 | 7 | 5 | 6 | 6 | 5 | 2005 | M | B | Female | - |
| 291 | 6 | 6 | 6 | 6 | 6 | 6 | 6 | 6 | 2005 | M | B | Female | - |
| 290 | 6 | 7 | 7 | 5 | 5 | 6 | 7 | 6 | 2005 | M | B | Male | - |
| 289 | 5 | 8 | 9 | 5 | 6 | 7 | 6 | 2 | 2005 | M | B | Male | - |
| 288 | 7 | 7 | 7 | 6 | 5 | 2 | 5 | NE | 2005 | M | B | Female | - |
| 287 | 5 | 6 | 7 | 5 | 4 | 2 | 3 | 2 | 2005 | M | B | Female | - |
| 286 | 5 | 8 | 8 | 5 | 2 | 6 | 4 | 3 | 2005 | M | B | Female | - |
| 285 | 7 | 9 | 8 | 8 | 8 | 7 | 7 | 5 | 2005 | M | B | Female | - |
| 284 | 5 | 7 | 7 | 3 | NE | 3 | 6 | 2 | 2005 | M | B | Female | - |
| 283 | 6 | 7 | 6 | 4 | 5 | 2 | NE | 3 | 2005 | M | B | Female | - |
| 282 | 8 | 7 | 7 | 6 | 5 | 4 | 7 | 2 | 2005 | M | B | Male | - |
| 281 | 7 | 8 | 9 | 9 | 9 | 7 | 6 | 6 | 2005 | M | B | Female | - |
| 280 | 5 | 7 | 6 | 6 | 6 | 6 | 6 | 6 | 2005 | M | B | Male | - |
| 279 | 5 | 5 | 5 | 4 | 5 | 2 | NE | 3 | 2005 | M | B | Female | - |
| 278 | 6 | 6 | 9 | 6 | 6 | 6 | 6 | 6 | 2005 | M | B | Female | - |
| 277 | 4 | 4 | 4 | 3 | 3 | 2 | NE | 4 | 2005 | M | B | Female | - |
| 276 | 6 | 6 | 6 | 6 | 6 | 5 | 6 | 6 | 2005 | M | B | Female | - |
| 275 | 6 | 9 | 7 | 5 | 3 | 4 | 3 | 5 | 2005 | M | B | Male | - |
| 274 | 6 | 6 | 6 | 6 | 6 | 7 | 6 | 5 | 2005 | M | A | Female | - |
| 273 | 5 | 7 | 5 | 2 | 5 | 7 | 6 | 2 | 2005 | M | A | Female | - |
| 272 | 9 | 8 | 7 | 8 | 10 | 8 | 9 | 5 | 2005 | M | A | Female | - |
| 271 | 9 | 8 | 9 | 8 | 8 | 8 | 8 | 6 | 2005 | M | A | Female | - |
| 270 | 5 | 6 | 5 | 6 | 7 | 8 | 5 | 6 | 2005 | M | A | Female | - |
| 269 | 5 | 6 | 8 | 6 | 7 | 8 | 6 | 5 | 2005 | M | A | Female | - |
| 268 | 6 | 8 | 6 | 6 | 7 | 9 | 7 | 5 | 2005 | M | A | Female | - |
| 267 | 8 | 6 | 5 | 7 | 7 | 6 | 6 | 6 | 2005 | M | A | Male | - |
| 266 | 7 | 8 | 9 | 10 | 9 | 8 | 9 | 5 | 2005 | M | A | Female | - |
| 265 | 6 | 5 | 4 | 5 | 8 | 7 | 6 | 6 | 2005 | M | A | Female | - |
| 264 | 8 | 7 | 8 | 8 | 10 | 8 | 6 | 7 | 2005 | M | A | Female | - |
| 263 | 6 | 6 | 6 | 7 | 8 | 7 | 6 | 6 | 2005 | M | A | Male | - |
| 262 | 7 | 6 | 5 | 5 | 6 | 7 | 6 | 5 | 2005 | M | A | Female | - |
| 261 | 6 | 6 | 5 | 6 | 6 | 7 | 6 | 5 | 2005 | M | A | Male | - |
| 260 | 7 | 6 | 6 | 7 | 9 | 6 | 6 | 6 | 2005 | M | A | Female | - |
| 259 | 6 | 5 | 4 | 2 | 5 | 6 | 3 | 3 | 2005 | M | A | Female | - |
| 258 | 5 | 6 | 6 | 5 | 6 | 7 | 5 | 6 | 2005 | M | A | Male | - |
| 257 | 4 | 5 | 5 | 8 | 7 | 5 | 8 | 6 | 2004 | E | A | Female | - |
| 256 | 5 | 8 | 8 | 8 | 8 | 7 | 8 | 8 | 2004 | E | A | Female | - |
| 255 | 4 | 3 | 3 | 4 | 4 | 3 | 7 | 5 | 2004 | E | A | Female | - |
| 254 | 4 | 5 | 5 | 3 | 5 | 5 | 6 | 5 | 2004 | E | A | Female | - |
| 253 | 6 | 8 | 5 | 8 | 5 | 4 | 8 | 6 | 2004 | E | A | Male | - |
| 252 | 8 | 8 | 8 | 9 | 8 | 6 | 10 | 8 | 2004 | E | A | Female | - |
| 251 | NE | NE | NE | NE | NE | NE | NE | 4 | 2004 | E | A | Female | - |
| 250 | NE | NE | NE | NE | NE | NE | NE | 4 | 2004 | E | A | Female | - |
| 249 | NE | 5 | 3 | 2 | 3 | 3 | 7 | 5 | 2004 | E | A | Male | - |
| 248 | 4 | 6 | 7 | 7 | 7 | 6 | 6 | 8 | 2004 | E | A | Female | - |
| 247 | 5 | 9 | 7 | 8 | 7 | 8 | 8 | 8 | 2004 | E | A | Female | - |
| 246 | 4 | 6 | 7 | 8 | 7 | 5 | 8 | 5 | 2004 | E | A | Male | - |
| 245 | 7 | 5 | 7 | 5 | 6 | 5 | 7 | 5 | 2004 | E | A | Female | - |
| 244 | 7 | 5 | 4 | 2 | 5 | 5 | 8 | 5 | 2004 | E | A | Female | - |
| 243 | 4 | 5 | 5 | 6 | 6 | 5 | 7 | 6 | 2004 | E | A | Female | - |
| 242 | 4 | 5 | 5 | 3 | 4 | 2 | 8 | 5 | 2004 | E | A | Female | - |
| 241 | 5 | 5 | 5 | 5 | 5 | 3 | 8 | 6 | 2004 | E | A | Female | - |
| 240 | 4 | 5 | 5 | 2 | 4 | 5 | 8 | 5 | 2004 | E | A | Female | - |
| 239 | 6 | 5 | 6 | 6 | 5 | 6 | 6 | 6 | 2004 | E | A | Female | - |
| 238 | 8 | 6 | 4 | 7 | 7 | 5 | 8 | 7 | 2004 | E | A | Male | - |
| 237 | 4 | 6 | 6 | 8 | 7 | 6 | 6 | NE | 2004 | M | B | Female | - |
| 236 | NE | 5 | NE | NE | NE | NE | 3 | NE | 2004 | M | B | Female | - |
| 235 | 5 | 6 | 4 | NE | 2 | 5 | 4 | 2 | 2004 | M | B | Female | - |
| 234 | 7 | 8 | 7 | 6 | 7 | 7 | 5 | 6 | 2004 | M | B | Female | - |
| 233 | 5 | 3 | NE | NE | 2 | 3 | NE | NE | 2004 | M | B | Female | - |
| 232 | 6 | 6 | 6 | 7 | 6 | 7 | 6 | 6 | 2004 | M | B | Male | - |
| 231 | 5 | 6 | 6 | 8 | 7 | 5 | 8 | 5 | 2004 | M | B | Male | - |
| 230 | 3 | 3 | NE | NE | NE | 2 | 2 | 2 | 2004 | M | B | Male | - |
| 229 | 6 | 5 | 3 | 5 | 3 | 5 | 5 | 5 | 2004 | M | B | Male | - |
| 228 | 7 | 8 | 8 | 8 | 9 | 8 | 7 | 6 | 2004 | M | B | Female | - |
| 227 | 5 | 7 | 7 | 3 | 2 | 2 | 3 | 6 | 2004 | M | B | Female | - |
| 226 | 8 | 7 | 7 | 5 | 7 | 6 | 5 | 6 | 2004 | M | B | Female | - |
| 225 | 6 | 6 | 5 | 6 | 6 | 6 | 6 | 5 | 2004 | M | B | Female | - |
| 224 | 5 | 5 | 3 | 5 | 6 | 6 | 6 | NE | 2004 | M | B | Female | - |
| 223 | 6 | 7 | 8 | 6 | 7 | 6 | 8 | 5 | 2004 | M | B | Male | - |
| 222 | 6 | 5 | 6 | 5 | 5 | 6 | 6 | 5 | 2004 | M | B | Female | - |
| 221 | 6 | 6 | 6 | 6 | 6 | 6 | 6 | 5 | 2004 | M | B | Male | - |
| 220 | 6 | 7 | 7 | NE | 2 | 4 | 5 | 3 | 2004 | M | B | Male | - |
| 219 | 10 | 10 | 10 | 9 | 10 | 9 | 10 | 9 | 2004 | M | B | Female | - |
| 218 | NE | 6 | 4 | 5 | 6 | 6 | 6 | 6 | 2004 | M | B | Male | - |
| 217 | 5 | 5 | 4 | 4 | 6 | 3 | 6 | NE | 2004 | M | B | Male | - |
| 216 | 8 | 8 | 8 | 5 | 7 | 7 | 5 | 7 | 2004 | M | B | Female | - |
| 215 | 6 | 5 | 5 | 5 | 5 | 5 | 5 | NE | 2004 | M | A | Female | - |
| 214 | 3 |  | 6 | 6 | 5 |  | 6 | 5 | 2004 | M | A | Female | - |
| 213 | 5 | 6 | 6 | 6 | 6 | 6 | 6 | 5 | 2004 | M | A | Female | - |
| 212 | 5 | 7 | 5 | 5 | 5 | 2 | 9 | NE | 2004 | M | A | Female | - |
| 211 | 6 | 4 | 5 | NE | 5 | 2 | 5 | NE | 2004 | M | A | Female | - |
| 210 | 2 | 4 | 5 | NE | 3 | 2 | 5 | 2 | 2004 | M | A | Female | - |
| 209 | 6 | 7 | 6 | 6 | 7 | 6 | 5 | 6 | 2004 | M | A | Female | - |
| 208 | 8 | 6 | 6 | 6 | 7 | 6 | 6 | 5 | 2004 | M | A | Male | - |
| 207 | 6 | 5 | 4 | 5 | 4 | 2 | 5 | NE | 2004 | M | A | Female | - |
| 206 | 2 | 3 | 2 | 3 | 4 | 2 | 3 | NE | 2004 | M | A | Female | - |
| 205 | 5 | 5 | 6 | 6 | 6 | 5 | 6 | 5 | 2004 | M | A | Male | - |
| 204 | 5 | 6 | 6 | 2 | 4 | 6 | 6 | 5 | 2004 | M | A | Female | - |
| 203 | 4 | 6 | 6 | 6 | 8 | 5 | 6 | 5 | 2004 | M | A | Female | - |
| 202 | 6 | 6 | 6 | 6 | 6 | 5 | 6 | 5 | 2004 | M | A | Male | - |
| 201 | 3 | 5 | 5 | NE | 6 | 2 | 2 | 2 | 2004 | M | A | Female | - |
| 200 | 6 | 8 | 7 | 9 | 9 | 8 | 8 | 6 | 2004 | M | A | Male | - |
| 199 | 6 | 7 | 5 | 6 | 5 | 5 | 6 | NE | 2004 | M | A | Female | - |
| 198 | 7 | 7 | 6 | 6 | NE | 6 | 7 | NE | 2004 | M | A | Female | - |
| 197 | 2 | 3 | 2 | NE | 2 |  | 2 | NE | 2004 | M | A | Female | - |
| 196 | 5 | 7 | 6 | 8 | 7 | 2 | 9 | 5 | 2004 | M | A | Female | - |
| 195 | W | 6 | NE | W | W | 5 | 5 | W | 2004 | M | A | Male | - |
| 194 | 6 | 5 | 6 | 8 | 5 | 2 | 5 | NE | 2004 | M | A | Female | - |
| 193 | 4 | 5 | 2 | NE | 4 | 5 | 6 | 3 | 2004 | M | A | Female | - |
| 192 | 5 | 6 | 6 | NE | 4 | 5 | 6 | NE | 2004 | M | A | Female | - |
| 191 |  | 4 |  |  | 2 | 2 | 3 | 5 | 2003 | E | A | Female | - |
| 190 | 6 | 6 | NE | NE | NE | 6 | NE | NE | 2003 | E | A | Male | - |
| 189 | 7 | 7 | 9 | 9 | 9 | 7 | 8 | 9 | 2003 | E | A | Female | - |
| 188 | 7 | 7 | 8 | 8 | 8 | 9 | 7 | 9 | 2003 | E | A | Female | - |
| 187 | 6 | 7 | 8 | 9 | 8 | 7 | 3 | 9 | 2003 | E | A | Female | - |
| 186 | 6 | 6 | 2 | 6 | 6 | 6 | 3 | 6 | 2003 | E | A | Female | - |
| 185 | 6 | 6 | NE | 3 | 5 | 6 | NE | 5 | 2003 | E | A | Male | - |
| 184 | 6 | 5 | 5 | NE | 6 | 6 | 7 | 5 | 2003 | E | A | Male | - |
| 183 | 8 | 6 | 5 | 7 | 7 | 5 | 6 | 6 | 2003 | E | A | Female | - |
| 182 | NE | 6 | 6 | 6 | 6 | NE | 6 | 6 | 2003 | E | A | Male | - |
| 181 | 7 | 8 | 8 | 7 | 6 | 7 | 9 | 8 | 2003 | E | A | Female | - |
| 180 | 5 | 7 | 9 | 6 | 7 | 5 | 9 | 9 | 2003 | E | A | Female | - |
| 179 | 5 | 5 | 4 | 5 | 3 | 2 | 4 | 7 | 2003 | E | A | Female | - |
| 178 | 6 | 7 | 7 | 5 | 7 | 5 | 8 | 7 | 2003 | E | A | Female | - |
| 177 | 6 | 5 | 5 | 5 | 5 | 5 | 6 | 5 | 2003 | E | A | Male | - |
| 176 | 6 | 5 | 6 | 8 | 4 | 5 | 5 | 8 | 2003 | E | A | Female | - |
| 175 | 6 | 8 | 8 | 7 | 8 | 6 | 8 | 9 | 2003 | E | A | Female | - |
| 174 | NE | NE | NE | NE | NE | NE | NE | NE | 2003 | E | A | Female | - |
| 173 | 2 | 6 | 2 | NE | NE | NE | 2 | 2 | 2003 | E | A | Female | - |
| 172 | 6 | 4 |  | NE | NE | NE | 2 | NE | 2003 | E | A | Male | - |
| 171 | 3 | 3 | 3 | 3 | 3 | NE | 3 | 5 | 2003 | E | A | Female | - |
| 170 | 6 | 7 | 6 | 6 | 7 | 5 | 6 | 7 | 2003 | E | A | Female | - |
| 169 | 7 | 3 | 2 | NE | NE | 5 | 2 | NE | 2003 | E | A | Female | - |
| 168 | 6 | 5 | 2 | NE | 4 | NE | 3 | NE | 2003 | E | A | Female | - |
| 167 | 4 | 3 | 8 | 2 | NE | 7 | 10 | NE | 2003 | M | B | Female | - |
| 166 | NE | NE | NE | NE | NE | NE | NE | NE | 2003 | M | B | Female | - |
| 165 | 8 | 7 | 7 | 5 | 6 | 6 | 7 | 5 | 2003 | M | B | Female | - |
| 164 | 8 | 6 | 5 | NE | NE | 8 | NE | NE | 2003 | M | B | Male | - |
| 163 | 5 | 6 | 5 | 6 | 5 | 6 | 6 | 6 | 2003 | M | B | Female | - |
| 162 | 9 | 9 | 10 | 9 | 5 | 7 | 10 | 7 | 2003 | M | B | Female | - |
| 161 | 9 | 6 | 9 | 8 | 5 | 8 | 10 | 3 | 2003 | M | B | Female | - |
| 160 | 8 | 10 | 10 | 10 | 9 | 9 | 10 | 9 | 2003 | M | B | Female | - |
| 159 | 6 | 6 | 6 | 6 | 6 | 7 | 6 | 7 | 2003 | M | B | Female | - |
| 158 | 8 | 9 | 10 | 9 | 9 | 7 | 10 | 7 | 2003 | M | B | Female | - |
| 157 | 4 | NE | NE | NE | NE | 3 | NE | NE | 2003 | M | B | Female | - |
| 156 | 6 | 5 | NE | NE | NE | 7 | 6 | NE | 2003 | M | B | Female | - |
| 155 | NE | NE | NE | NE | NE | NE | NE | NE | 2003 | M | B | Male | - |
| 154 | 6 | 5 | 6 | 7 | 6 | 7 | 6 | 5 | 2003 | M | B | Male | - |
| 153 | 6 | 3 | 8 | NE | NE | 6 | 9 | NE | 2003 | M | B | Female | - |
| 152 | 6 | 5 | 8 | 6 | 7 | 9 | 8 | 7 | 2003 | M | B | Female | - |
| 151 | 6 | 5 | 5 | NE | NE | 7 | 6 | 2 | 2003 | M | B | Male | - |
| 150 | 3 | 3 | NE | NE | NE | 2 | NE | NE | 2003 | M | B | Female | - |
| 149 | 6 | 6 | 6 | 6 | 6 | 8 | 6 | 6 | 2003 | M | B | Female | - |
| 148 | 6 | 3 | 5 | 2 | NE | 6 | 7 | 5 | 2003 | M | B | Female | - |
| 147 | 4 | 5 | NE | 2 | 2 | 6 | NE | 3 | 2003 | M | B | Male | - |
| 146 | 3 | 3 | NE | NE | 5 | 2 | 6 | 2 | 2003 | M | B | Male | - |
| 145 | 7 | 6 | 8 | 7 | 7 | 7 | 9 | 6 | 2003 | M | B | Female | - |
| 144 | 7 | 5 | 7 | 5 | NE | 8 | 9 | 5 | 2003 | M | B | Female | - |
| 143 | 9 | 6 | 7 | 5 | 5 | 7 | 8 | 5 | 2003 | M | B | Female | - |
| 142 | 9 | 6 | 6 | 7 | 7 | 6 | 8 | 7 | 2003 | M | B | Male | - |
| 141 | NE | NE | NE | NE | NE | NE | NE | NE | 2003 | M | B | Male | - |
| 140 | 5 | 6 | 8 | NE | 6 | 3 | 8 | NE | 2003 | M | A | Female | - |
| 139 | 4 | 5 | 8 | 6 | 6 | 7 | 7 | 5 | 2003 | M | A | Female | - |
| 138 | 4 | 5 | 9 | 6 | 6 | 6 | 7 | 5 | 2003 | M | A | Female | - |
| 137 | 6 | 7 | 5 | 5 | 6 | 7 | 8 | 6 | 2003 | M | A | Female | - |
| 136 | 4 | 5 | 8 | 5 | 3 | 3 | 7 | NE | 2003 | M | A | Female | - |
| 135 | 4 | 5 | 6 | 7 | 6 | 3 | 6 | NE | 2003 | M | A | Female | - |
| 134 | 4 | 5 | 9 | 6 | 4 | 3 | 9 | 2 | 2003 | M | A | Male | - |
| 133 | 6 | 5 | 5 | 2 | 6 | 4 | 6 | 3 | 2003 | M | A | Male | - |
| 132 | 4 | 5 | 5 | 8 | 7 | 5 | 6 | 5 | 2003 | M | A | Male | - |
| 131 | 8 | 9 | 10 | 9 | 10 | 10 | 9 | 8 | 2003 | M | A | Female | - |
| 130 | 8 | 7 | 9 | 7 | 8 | 9 | 9 | 8 | 2003 | M | A | Female | - |
| 129 | 4 | 3 | 5 | 6 | 3 | 3 | 6 | NE | 2003 | M | A | Male | - |
| 128 | 7 | 6 | 10 | 6 | 8 | 8 | 6 | 5 | 2003 | M | A | Female | - |
| 127 | 6 | 6 | 6 | 6 | NE | 2 | 4 | NE | 2003 | M | A | Female | - |
| 126 | 4 | 5 | 7 | 3 | NE | 3 | 9 | NE | 2003 | M | A | Female | - |
| 125 | NE | NE | NE | NE | NE | NE | NE | NE | 2003 | M | A | Male | - |
| 124 | 8 | 6 | 9 | 8 | 7 | 6 | 9 | 5 | 2003 | M | A | Female | - |
| 123 | 4 | 5 | 8 | 5 | NE | 3 | 8 | NE | 2003 | M | A | Female | - |
| 122 | 4 | 5 | 7 | 5 | 4 | 3 | 7 | 2 | 2003 | M | A | Male | - |
| 121 | NE | NE | NE | NE | NE | NE | NE | NE | 2003 | M | A | Female | - |
| 120 | NE | NE | NE | NE | NE | NE | NE | NE | 2003 | M | A | Female | - |
| 119 | 6 | 5 | 10 | 6 | 5 | 5 | 6 | 2 | 2003 | M | A | Female | - |
| 118 | 6 | 6 | 9 | 7 | 6 | 5 | 9 | 3 | 2003 | M | A | Female | - |
| 117 | 4 | 3 | 8 | 2 | 4 | 3 | 4 | 5 | 2003 | M | A | Female | - |
| 116 | 4 | 5 | 4 | 2 | NE | 3 | 8 | NE | 2003 | M | A | Female | - |
| 115 | 4 | 5 | 7 | 2 | NE | 3 | 7 | NE | 2003 | M | A | Female | - |
| 114 | 5 | 3 | 8 | 5 | 4 | 5 | 6 | NE | 2003 | M | A | Female | - |
| 113 | 6 | 6 | NE | NE | 2 | 7 | NE | 3 | 2002 | E | A | Male | - |
| 112 | 8 | 7 | 5 | 8 | 7 | 7 | 6 | 7 | 2002 | E | A | Male | - |
| 111 | 7 | 8 | 2 | 5 | 5 | 7 | NE | 6 | 2002 | E | A | Female | - |
| 110 | 5 | 5 | NE | NE | 3 | 5 | NE | 5 | 2002 | E | A | Male | - |
| 109 | 7 | 4 | NE | NE | 4 | 5 | 3 | 4 | 2002 | E | A | Male | - |
| 108 | 4 | 4 | NE | NE | NE | NE | 2 | 3 | 2002 | E | A | Male | - |
| 107 | 3 | 4 | NE | NE | 2 | 4 | 2 | 4 | 2002 | E | A | Female | - |
| 106 | 6 | 5 | 2 | 3 | 3 | NE | 6 | 4 | 2002 | E | A | Female | - |
| 105 | 7 | 7 | 6 | 5 | 6 | 5 | 7 | 7 | 2002 | E | A | Male | - |
| 104 | 9 | 7 | 9 | 8 | 8 | 6 | 6 | 7 | 2002 | E | A | Female | - |
| 103 | 6 | NE | NE | NE | NE | NE | 6 | NE | 2002 | E | A | Male | - |
| 102 | 4 | 4 | NE | NE | NE | 4 | 3 | 4 | 2002 | E | A | Male | - |
| 101 | NE | 6 | 6 | NE | NE | 6 | 6 | 6 | 2002 | E | A | Male | - |
| 100 | NE | NE | NE | NE | NE | NE | NE | NE | 2002 | E | A | Female | - |
| 99 | 8 | 6 | 5 | 7 | 6 | 7 | 6 | 7 | 2002 | E | A | Female | - |
| 98 | 4 | 4 | 2 | 2 | 2 | 3 | 3 | 4 | 2002 | E | A | Male | - |
| 97 | 4 |  | NE | NE | 2 | NE | NE | NE | 2002 | E | A | Female | - |
| 96 | 6 | 4 | NE | NE | 3 | 6 | 3 | 5 | 2002 | E | A | Female | - |
| 95 | 4 | 4 | 4 | 3 | 3 | 2 | 4 | 6 | 2002 | E | A | Female | - |
| 94 | 6 | 4 | n | NE | 3 | 6 | NE | 5 | 2002 | E | A | Female | - |
| 93 | 7 | 7 | 6 | 8 | 7 | 6 | 9 | 8 | 2002 | E | A | Female | - |
| 92 | 6 | 4 | NE | 2 | NE | 6 | 6 | 6 | 2002 | E | A | Male | - |
| 91 | NE | NE | NE | NE | NE | NE | NE | NE | 2002 | E | A | Male | - |
| 90 | NE | NE | NE | NE | NE | NE | NE | NE | 2002 | M | A | Female | - |
| 89 | 7 | 8 | 7 | 8 | 7 | 7 | 9 | 7 | 2002 | M | A | Male | - |
| 88 | 4 | 4 | 5 | 3 | 5 | 2 | 6 | 4 | 2002 | M | A | Female | - |
| 87 | 9 | 10 | 9 | 9 | 10 | 8 | 7 | 8 | 2002 | M | A | Male | - |
| 86 | NE | 2 | 2 | NE | NE | NE | 2 | 3 | 2002 | M | A | Female | - |
| 85 | 6 | 6 | 7 | 8 | 6 | 5 | 7 | 7 | 2002 | M | A | Male | - |
| 84 | 9 | 7 | 8 | 7 | 7 | 7 | 7 | 8 | 2002 | M | A | Female | - |
| 83 | NE | NE | NE | NE | NE | NE | NE | NE | 2002 | M | A | Female | - |
| 82 | 5 | 6 | 5 | 5 | 5 | 2 | 5 | 7 | 2002 | M | A | Female | - |
| 81 | 2 | 5 | 3 | 5 | 5 | 2 | 7 | 6 | 2002 | M | A | Male | - |
| 80 | 5 | 5 | 5 | 5 | 3 | 5 | 6 | 7 | 2002 | M | A | Female | - |
| 79 | NE | 6 | NE | NE | 6 | NE | 6 | 6 | 2002 | M | A | Female | - |
| 78 | NE | 4 | 3 | 3 | NE | 2 | 9 | 4 | 2002 | M | A | Male | - |
| 77 | 3 | 5 | 4 | 5 | 3 | 2 | 5 | 6 | 2002 | M | A | Female | - |
| 76 | 3 | 3 | 3 | 2 | NE | 2 | 5 | 3 | 2002 | M | A | Male | - |
| 75 | 3 | 5 | 4 | 5 | 3 | 2 | 5 | 3 | 2002 | M | A | Female | - |
| 74 | 5 | 7 | 5 | 5 | 4 | 2 | 3 | 4 | 2002 | M | A | Female | - |
| 73 | 6 | 10 | 9 | 9 | 10 | 8 | 8 | 8 | 2002 | M | A | Female | - |
| 72 | NE | 6 | 5 | 5 | 5 | NE | 5 | 4 | 2002 | M | A | Female | - |
| 71 | 2 | 6 | 5 | 7 | 6 | 2 | 7 | 6 | 2002 | M | A | Male | - |
| 70 | 6 | 5 | 6 | 5 | 5 | 7 | 6 | 6 | 2002 | M | A | Female | - |
| 69 | 5 | 7 | 7 | 7 | 7 | 6 | 7 | 6 | 2002 | M | A | Female | - |
| 68 | NE | NE | 2 | NE | NE | NE | NE | 2 | 2002 | M | A | Female | - |
| 67 | 6 | 4 | 4 | 5 | 3 | 2 | 5 | 3 | 2002 | M | A | Female | - |
| 66 | 3 | 5 | 5 | 5 | 6 | 2 | 5 | 7 | 2002 | M | A | Female | - |
| 65 | NE | NE | n | NE | NE | NE | NE | NE | 2002 | M | A | Female | - |
| 64 | 8 | 8 | 9 | 9 | 10 | 9 | 9 | 8 | 2002 | M | A | Female | - |
| 63 | 3 | 2 | 2 | 3 | 2 | 2 | 5 | 4 | 2002 | M | A | Female | - |
| 62 | NE | NE | 2 | NE | NE | NE | 2 | 3 | 2002 | M | A | Female | - |
| 61 | 6 | 6 | 6 | 6 | NE | NE | 6 | 6 | 2002 | M | A | Male | - |
| 60 | 7 | 8 | 7 | 8 | 6 | 8 | 6 | 8 | 2001 | E | A | Female | - |
| 59 | 6 | 6 | 5 | 5 | 5 | 5 | 7 | 5 | 2001 | E | A | Female | - |
| 58 | 6 | 8 | 8 | 8 | 6 | 6 | 6 | 6 | 2001 | E | A | Male | - |
| 57 | 6 | 7 | 5 | 9 | 7 | 5 | 8 | 6 | 2001 | E | A | Male | - |
| 56 | NE | NE | NE | NE | NE | NE | NE | NE | 2001 | E | A | Female | - |
| 55 | 4 | NE | 3 | NE | NE | NE | 5 | 2 | 2001 | E | A | Male | - |
| 54 | 5 | 4 | 5 | 6 | 5 | 5 | 6 | 5 | 2001 | E | A | Female | - |
| 53 | 6 | 6 | 6 | 6 | 7 | 6 | 6 | 6 | 2001 | E | A | Female | - |
| 52 | NE | NE | NE | NE | NE | NE | NE | NE | 2001 | E | A | Female | - |
| 51 | 6 | 7 | 5 | 6 | 5 | 7 | 7 | 6 | 2001 | E | A | Female | - |
| 50 | 5 | 4 | 3 | 3 | NE | 4 | 6 | 2 | 2001 | E | A | Female | - |
| 49 | 6 | 6 | 3 | 3 | 5 | 5 | 7 | 5 | 2001 | E | A | Male | - |
| 48 | 8 | 8 | 8 | 8 | 5 | 8 | 7 | 7 | 2001 | E | A | Female | - |
| 47 | 6 | 6 | 6 | 6 | NE | NE | 6 | 6 | 2001 | E | A | Male | - |
| 46 | 5 | 8 | 6 | 6 | 5 | 6 | 6 | 6 | 2001 | E | A | Female | - |
| 45 | 7 | 6 | 6 | 6 | 6 | 6 | 6 | 6 | 2001 | E | A | Male | - |
| 44 | 5 | 7 | 6 | 8 | 6 | 7 | 7 | 5 | 2001 | E | A | Female | - |
| 43 | 6 | 4 | NE | 2 | NE | 4 | 7 | 2 | 2001 | E | A | Male | - |
| 42 | 5 | 5 | 5 | 5 | 5 | 6 | 7 | 6 | 2001 | E | A | Male | - |
| 41 | 4 | 6 | 6 | 2 | 3 | 6 | 6 | 5 | 2001 | E | A | Male | - |
| 40 | 5 | 7 | 8 | 7 | 6 | 7 | 6 | 6 | 2001 | E | A | Female | - |
| 39 | 5 | 8 | 7 | 7 | 7 | 7 | 6 | 6 | 2001 | E | A | Female | - |
| 38 | 6 | 5 | 2 | 2 | 2 | 4 | 8 | 5 | 2001 | E | A | Male | - |
| 37 | NE | NE | NE | NE | NE | NE | NE | NE | 2001 | E | A | Male | - |
| 36 | NE | NE | NE | NE | NE | NE | NE | NE | 2001 | E | A | Female | - |
| 35 | 4 | 5 | 3 | 3 | 4 | 5 | 5 | 3 | 2001 | E | A | Male | - |
| 34 | 7 | 6 | 6 | 5 | 6 | 5 | 6 | 6 | 2001 | E | A | Male | - |
| 33 | NE | NE | NE | NE | NE | NE | NE | NE | 2001 | E | A | Female | - |
| 32 | 5 | 8 | 7 | 8 | 6 | 8 | 6 | 6 | 2001 | E | A | Female | - |
| 31 | 5 | 4 | 2 | 2 | NE | 6 | 8 | 5 | 2001 | E | A | Male | - |
| 30 | 6 | 6 | 6 | 6 | NE | NE | 6 | 6 | 2001 | E | A | Male | - |
| 29 | NE | NE | NE | NE | NE | NE | NE | NE | 2001 | M | A | Female | - |
| 28 | 5 | 7 | 7 | 5 | 7 | 7 | 7 | 5 | 2001 | M | A | Female | - |
| 27 | 5 | 6 | 6 | 6 | 6 | 7 | 7 | 5 | 2001 | M | A | Female | - |
| 26 | NE | NE | NE | NE | NE | NE | NE | NE | 2001 | M | A | Female | - |
| 25 | NE | NE | NE | NE | NE | NE | NE | NE | 2001 | M | A | Male | - |
| 24 | NE | NE | NE | NE | NE | NE | NE | NE | 2001 | M | A | Female | - |
| 23 | 5 | 6 | 7 | 6 | 6 | 6 | 6 | 7 | 2001 | M | A | Male | - |
| 22 | NE | NE | NE | NE | NE | NE | NE | NE | 2001 | M | A | Female | - |
| 21 | NE | NE | NE | NE | NE | NE | NE | NE | 2001 | M | A | Female | - |
| 20 | 4 | 5 | 4 | 3 | 5 | 3 | 7 | 5 | 2001 | M | A | Female | - |
| 19 | NE | NE | NE | NE | NE | NE | NE | NE | 2001 | M | A | Male | - |
| 18 | 6 | 6 | 6 | 6 | 6 | 6 | 6 | 6 | 2001 | M | A | Female | - |
| 17 | 4 | 3 | NE | 3 | 3 | 3 | 4 | 5 | 2001 | M | A | Female | - |
| 16 | 10 | 9 | 9 | 7 | 9 | 8 | 9 | 6 | 2001 | M | A | Female | - |
| 15 | NE | NE | NE | NE | NE | NE | NE | NE | 2001 | M | A | Female | - |
| 14 | 9 | 10 | 10 | 10 | 9 | 9 | 10 | 9 | 2001 | M | A | Female | - |
| 13 | NE | NE | NE | 6 | 6 | NE | NE | 6 | 2001 | M | A | Female | - |
| 12 | 7 | 6 | NE | 5 | 5 | 6 | 7 | 5 | 2001 | M | A | Male | - |
| 11 | NE | NE | NE | NE | NE | NE | NE | NE | 2001 | M | A | Male | - |
| 10 | NE | NE | NE | NE | NE | NE | NE | NE | 2001 | M | A | Male | - |
| 9 | 5 | 5 | 6 | 5 | 7 | 7 | 6 | 6 | 2001 | M | A | Female | - |
| 8 | NE | NE | NE | NE | NE | NE | NE | NE | 2001 | M | A | Female | - |
| 7 | 8 | 5 | 6 | 5 | 6 | 7 | 8 | 7 | 2001 | M | A | Male | - |
| 6 | 6 | 6 | 7 | 7 | 7 | 6 | 7 | 6 | 2001 | M | A | Female | - |
| 5 | NE | NE | NE | NE | NE | NE | NE | NE | 2001 | M | A | Female | - |
| 4 | NE | NE | NE | NE | NE | NE | NE | NE | 2001 | M | A | Male | - |
| 3 | 5 | 5 | 5 | 5 | 5 | 5 | 7 | 5 | 2001 | M | A | Female | - |
| 2 | 5 | 8 | 8 | 6 | 7 | 6 | 7 | 6 | 2001 | M | A | Female | - |
| 1 | 4 | 3 | NE | 3 | 2 | 3 | 4 | 3 | 2001 | M | A | Female | - |

ID: student identification number, S1: subject 1 (*"Comunicación"*), S2: subject 2 *("Compraventa")*,

S3: subject 3 (*"Personal"*), S4: subject 4 *("Contabilidad")*, S5: subject 5 (*"SEFI"*), S6: subject 6 (*"Pública"*), S7: subject 7 (*"Informática"*), S8: subject 8 (*"FOL"*), D: distance, M: morning-early afternoon, A: late afternoon-evening, NE: not evaluated (the student did not attend the exams), W: the student formally withdrew from the subject before exam time.
